# *In meso in situ* serial X-ray crystallography of soluble and membrane proteins at cryogenic temperatures

**DOI:** 10.1107/S2059798315021683

**Published:** 2016-01-01

**Authors:** Chia-Ying Huang, Vincent Olieric, Pikyee Ma, Nicole Howe, Lutz Vogeley, Xiangyu Liu, Rangana Warshamanage, Tobias Weinert, Ezequiel Panepucci, Brian Kobilka, Kay Diederichs, Meitian Wang, Martin Caffrey

**Affiliations:** a Membrane Structural and Functional Biology Group, School of Medicine and School of Biochemistry and Immunology, Trinity College, Dublin 2, D02 R590, Ireland; b Swiss Light Source, Paul Scherrer Institute, CH-5232 Villigen, Switzerland; c Laboratory of Structure and Function of Biological Membranes, Center for Structural Biology and Bioinformatics, Université Libre de Bruxelles, 1050 Brussels, Belgium; d School of Medicine, Tsinghua University, Beijing 100084, People’s Republic of China; e Department of Molecular and Cellular Physiology, Stanford University School of Medicine, Stanford, CA 94305, USA; f Fachbereich Biologie, Universität Konstanz, Box 647, D-78457 Konstanz, Germany

**Keywords:** AlgE, β_2_-adrenergic receptor, bromine SAD, cubic phase, DgkA, experimental phasing, GPCR, *in meso*, *in situ*, insulin, kinase, lipid cubic phase, membrane protein, mesophase, PepT_St_, serial crystallography, sponge phase, sulfur SAD, transporter

## Abstract

Here, a method for presenting crystals of soluble and membrane proteins growing in the lipid cubic or sponge phase for *in situ* diffraction data collection at cryogenic temperatures is introduced. The method dispenses with the need for the technically demanding and inefficient crystal-harvesting step that is an integral part of the lipid cubic phase or *in meso* method of growing crystals. Crystals are dispersed in a bolus of mesophase sandwiched between thin plastic windows. The bolus contains tens to hundreds of crystals, visible with an in-line microscope at macromolecular crystallography synchrotron beamlines and suitably disposed for conventional or serial crystallographic data collection. Wells containing the crystal-laden boluses are removed individually from hermetically sealed glass plates in which crystallization occurs, affixed to pins on goniometer bases and excess precipitant is removed from around the mesophase. The wells are snap-cooled in liquid nitrogen, stored and shipped in Dewars, and manually or robotically mounted on a goniometer in a cryostream for diffraction data collection at 100 K, as is performed routinely with standard, loop-harvested crystals. The method is a variant on the recently introduced *in meso in situ* serial crystallography (IMISX) method that enables crystallo­graphic measurements at cryogenic temperatures where crystal lifetimes are enormously enhanced whilst reducing protein consumption dramatically. The new approach has been used to generate high-resolution crystal structures of a G-protein-coupled receptor, α-helical and β-barrel transporters and an enzyme as model integral membrane proteins. Insulin and lysozyme were used as test soluble proteins. The quality of the data that can be generated by this method was attested to by performing sulfur and bromine SAD phasing with two of the test proteins.

## Abstract 2

A method for performing high-throughput *in situ* serial X-ray crystallography with soluble and membrane proteins in the lipid cubic phase at cryogenic temperatures (100 K) is described. It works with nanogram to single-digit microgram quantities of protein and lipid (and ligand when present), and is compatible with both high-resolution native data collection and experimental phasing without the need for crystal harvesting.

## 1. Introduction   

The lipid cubic phase (LCP) or *in meso* method for crystallizing membrane proteins has delivered over 290 structures of integral membrane proteins (Caffrey, 2015 ▸). This represents about 12% of the membrane-protein structures in the Protein Data Bank (PDB). Importantly, half of these PDB records have been deposited in the last two years, indicating that the method is experiencing explosive growth. It is responsible for some of the highest profile structures in the field, particularly in the area of G-protein-coupled receptors (GPCRs). Further, the method works with a wide range of membrane-protein types, sizes and sources, including transporters, channels, peptides and enzymes. Soluble proteins can also be crystallized in the lipid cubic phase. A recent application for the mesophase is as a medium in which to perform injector-based serial crystallography with free-electron lasers (FELs; Liu *et al.*, 2013 ▸; Caffrey *et al.*, 2014 ▸; Weierstall *et al.*, 2014 ▸; Fenalti *et al.*, 2015 ▸; Kang *et al.*, 2015 ▸; Li *et al.*, 2015 ▸) and synchrotron X-rays (Botha *et al.*, 2015 ▸; Nogly *et al.*, 2015 ▸).

The *in meso* method makes use of a lipid liquid crystal or mesophase into the bilayer of which the membrane protein is reconstituted as a prelude to crystallogenesis. The mesophase itself is sticky and viscous and can be challenging to manipulate (Caffrey & Cherezov, 2009 ▸). Because the hosting mesophase in which crystallogenesis occurs is sensitive to hydration, trials are typically set up in watertight glass sandwich plates (Cherezov *et al.*, 2004 ▸). Opening the plates to harvest crystals requires the use of a glass-cutting tool along with considerable manual dexterity, good hand–eye coordination and patience (Li *et al.*, 2012 ▸). The harvesting process can be slow and inefficient, with the loss of valuable crystals. To obviate the need to harvest, we introduced a method for performing direct *in meso in situ* serial crystallographic (IMISX) measurements at room temperature (RT; Huang *et al.*, 2015 ▸). Special double-sandwich IMISX plates were developed for this purpose. These consist of an internal plate in which the protein-laden mesophase sits surrounded by precipitant solution and sandwiched between thin (25 µm) cyclic olefin copolymer (COC) films. The external sandwich is composed of glass plates which provide the watertight seal. The IMISX method worked spectacularly well, providing structures of alginate (AlgE) and peptide transporters (PepT_St_) as model integral membrane proteins and of lysozyme as a test soluble protein. Structures were solved by molecular replacement (MR) and by experimental phasing using bromine and native sulfur single-wavelength anomalous diffraction (SAD) methods to resolutions ranging from 1.8 to 2.8 Å with single-digit microgram quantities of protein. That sulfur SAD phasing worked is a testament to the exceptional quality of IMISX diffraction data.

The IMISX method was primarily developed for diffraction data collection at ambient or room temperature on macromolecular crystallography (MX) synchrotron beamlines. While the method worked well with a variety of proteins, it failed to deliver with others. One of the principal problems was radiation damage, which is severe at RT (Holton, 2009 ▸; Warkentin *et al.*, 2013 ▸). Thus, for suitably sized and well diffracting crystals, small angular wedges of relatively damage-free data could be collected from individual crystals. By repeating the process in a serial fashion on randomly oriented crystals in the mesophase, complete data sets of superior quality could be collected. However, this approach did not work with weakly diffracting and radiation-sensitive crystals. A case in point was the β_2_-adrenoreceptor (β_2_AR), where diffraction to no better than 4 Å resolution could be obtained by the IMISX method at RT. An obvious solution was to perform the IMISX measurements at the standard cryogenic temperature of 100 K, as implemented in this study.

Another shortcoming of the IMISX method was that it could not be simply implemented with membrane-protein crystals that grow optimally at temperatures other than RT. With such samples, collecting data at RT is not an option because the hosting mesophase changes with temperature (Caffrey, 2015 ▸) and crystals can be damaged in the process. Since most beamlines only provide for measurements either at cryogenic temperatures or at RT, this type of protein sample cannot be accommodated conveniently. One solution to this problem, as implemented in this study, is to harvest the IMISX sample at its optimal growth temperature and to snap-cool it in liquid nitrogen for storage and subsequent use in data collection conveniently at 100 K.

Yet another issue with the original IMISX method concerned the fact that crystals have varied lifetimes at RT. It is necessary therefore to either collect diffraction data when the crystal quality is at an optimum or to harvest and store the crystals at cryogenic temperatures until beamtime becomes available. The first option requires on-demand access to a synchrotron or an FEL X-ray source, which is not realistic. The second option is more practical and is implemented here.

The new method introduced in this report amounts to performing IMISX on intact, crystal-laden mesophase boluses in thin plastic wells that have been snap-cooled in liquid nitrogen. The wells are conveniently mounted individually on standard crystal-mounting pins on goniometer bases, stored in pucks and Dewars for positioning in a cryostream for diffraction data collection, either manually or by robot, essentially as for loop-harvested crystals. We refer to this as the IMISXcryo method.

That the IMISXcryo method works is demonstrated by structures determined to high resolution for four integral membrane proteins. These include β_2_AR, AlgE, PepT_St_ and diacylglycerol kinase (DgkA). Insulin and lysozyme were included as test soluble proteins. Experimental phasing by sulfur and bromine SAD was demonstrated with insulin and lysozyme, respectively, attesting to the quality of the data forthcoming by the IMISXcryo method. In several instances, complete data sets were collected from single or multiple crystals in a single well without the need for the complicated, laborious and inefficient harvesting of individual crystals. This highlights the many benefits of the method, which can be implemented readily in any membrane-protein laboratory performing *in meso* crystallogenesis and with access to a synchrotron or FEL X-ray source.

## 2. Materials and methods   

### 2.1. Materials   

Monoolein (9.9 MAG; M239-F4-X) was purchased from Nu-Chek Prep, Elysian, Minnesota, USA and 7.8 MAG (smm48 and TasC42) was synthesized and purified in-house following established procedures (Coleman *et al.*, 2004 ▸; Caffrey *et al.*, 2009 ▸). Cholesterol (catalogue No. C8667, lot 080M5304V), chicken egg-white lysozyme (catalogue No. L6876, lot SLBG8654V), porcine pancreas insulin (catalogue No. I5523, lot SLBH2842V), HEPES (catalogue No. H4034, lot SLBF8768V), sodium acetate (catalogue No. S2889, lot 079K0122), sodium phosphate (catalogue No. S9763, lot BCBF5244V), magnesium acetate (catalogue No. M0631, lot 069K03391), 1,4-butanediol (catalogue No. 49373-2, lot S22915-264), bis-tris (catalogue No. B9754, lot 118K5420), sodium sulfate (catalogue No. 23931-3, lot 066K0083), PEG 400 (catalogue No. 81172, lot BCBL5307V), hexylene glycol (2-methyl-2,4-pentanediol, MPD; catalogue No. 68340, lot BCBM6109V), sodium bromide (catalogue No. 71329, lot 132896), hydrochloric acid (catalogue No. 07102, lot SZBL2500V), ammonium phosphate dibasic (catalogue No. 09839, lot BCBK1426V) and ammonium phosphate monobasic (catalogue No. 216003, lot MKBJ9529V) were obtained from Sigma, St Louis, Missouri, USA. Sodium chloride (catalogue No. BP358-212, lot 132896) was sourced from Fisher Scientific, Loughborough, England. Potassium thiocyanate (catalogue No. HR2-245, lot 224514) was purchased from Hampton Research, Aliso Viejo, California, USA. The dipeptide H-Ala-Phe-OH (catalogue No. G1320.0001, lot 1047671) was obtained from BACHEM, Bubendorf, Switzerland. Di-mannuronate (catalogue No. OD45238, lot MD452381401) was from Carbosynth, Berkshire, England. Sodium citrate pH 5.6 (catalogue No. HR2-935-15, lot 214560) and microsyringes (catalogue No. 81030) were sourced from Hamilton, Bonaduz, Switzerland. Cyclic olefin copolymer (COC; catalogue No. TOPAS 8007) and harvesting cryoloops (catalogue Nos. M2-L18SP-20, M2-L18SP-30 and M2-L18SP-50) were from MiTeGen, Ithaca, New York, USA. The gonio­meter base [catalogue No. MD7-400, CryoCap (SPINE standard)] was purchased from Molecular Dimensions, Florida, USA. Standard glass (127.8 × 85.5 mm, 1 mm thick; catalogue No. 1527127092, lot 29642819) and No. 1.5 glass (124 × 84 mm, 0.15 mm thick; catalogue No. 01029990933, lot 30129819) were obtained from Marienfeld, Lauda-Königshofen, Germany. Perforated double-stick spacer tape (112 × 77 mm, 140 µm thick), perforation diameters (6 mm; catalogue No. 9500PC) and double-stick gasket (2 mm wide and 140 µm thick with outer dimensions 118 × 83 mm and inner dimensions 114 × 79 mm; catalogue No. TRI-9500PC) were purchased from Saunders, St Paul, Minnesota, USA. Glass-cutting tools (TCT Scriber & Glass Cutter, catalogue No. 633657) were obtained from Silverline, Yeovil, England. Rain-X rain repellent (catalogue No. 80199200, lot 5026349013414) was from Shell Car Care, Altrincham, England.

### 2.2. Methods   

#### 2.2.1. Protein-laden mesophase, manual and robot-based crystallization   

Six proteins were used in this study as follows: chicken egg-white lysozyme, porcine pancreas insulin, the alginate transporter AlgE from *Pseudomonas aeruginosa* PA01, a peptide transporter (PepT_St_) from *Streptococcus thermophilus*, a T4 lysozyme-β_2_-adrenergic receptor fusion (β_2_AR) and diacylglycerol kinase (DgkA) from *Escherichia coli* K-12. Complexes with dimannuronate, Ala-Phe (AF) and carazolol were used for AlgE, PepT_St_ and β_2_AR, respectively. Lysozyme and insulin were obtained from a commercial source and were used as received. AlgE, PepT_St_, β_2_AR and DgkA (Δ7 construct) were produced recombinantly and purified from biomass following published protocols (Tan *et al.*, 2014 ▸; Lyons *et al.*, 2014 ▸; Cherezov *et al.*, 2007 ▸; Li *et al.*, 2013 ▸). For use in *in meso* crystallization trials, lysozyme-laden and insulin-laden mesophase was produced by homogenizing two volumes of protein solution at 50 and 35 mg ml^−1^, respectively, in Milli-Q water and insulin buffer (10 m*M* EDTA, 50 m*M* Na_2_HPO_4_ pH 10.8), respectively, with three volumes of the monoacylglycerol (MAG) lipid monoolein (9.9 MAG) in a coupled-syringe mixing device (Cheng *et al.*, 1998 ▸) at 20°C as described by Caffrey & Cherezov (2009 ▸). A similar protocol was used for AlgE, PepT_St_, β_2_AR and DgkA, with the exception that for PepT_St_, AlgE and DgkA the hosting lipid used was 7.8 MAG, the lipid:protein solution ratio was 1 and the concentration of the protein solution was 10–12 mg ml^−1^. The protein-laden mesophase was dispensed into wells on crystallization plates manually or robotically at 293 K using 30–200 nl mesophase and 800–1000 nl precipitant solution, as described by Caffrey & Cherezov (2009 ▸). The robots used included instruments provided by Sias (XANTUS; Cherezov *et al.*, 2004 ▸), TTP Labtech (Mosquito LCP) and Art Robbins (Gryphon LCP; Li *et al.*, 2012 ▸). The precipitant solutions used with lysozyme consisted of 0.5–1 *M* NaBr, 50–100 m*M* sodium acetate pH 4.5, 15–30%(*v*/*v*) PEG 400. *In meso*-grown lysozyme crystals dissolved over the course of 2–4 d (Aherne *et al.*, 2012 ▸). They were longer-lived, providing more handling flexibility, when grown at the lower precipitant ingredient concentrations. Brominated lysozyme crystals were used for native and bromine SAD data collection. The precipitant used with insulin consisted of 0.1–0.2 *M* sodium phosphate pH 5.5–6.1, 33–38%(*w*/*v*) PEG 400. The precipitant solutions used with the membrane proteins were as follows: AlgE, 100 m*M* MES pH 6, 400 m*M* KSCN, 20%(*v*/*v*) PEG 400, 20–50 m*M* dimannuronate; PepT_St_, 250–325 m*M* NH_4_H_2_PO_4_, 100 m*M* HEPES pH 7.0, 21–22%(*v*/*v*) PEG 400, 10 m*M* Ala-Phe; β_2_AR, 30–35%(*v*/*v*) PEG 400, 0.1–0.2 *M* Na_2_SO_4_, 0.1 *M* bis-tris propane pH 6.5–7.0, 5–7%(*v*/*v*) 1,4-butanediol; DgkA, 3–6%(*v*/*v*) MPD, 0.1 *M* NaCl, 60 m*M* magnesium acetate, 50 m*M* trisodium citrate pH 5.6. With the exception of DgkA, where the crystals were grown at 277 K, all other proteins were crystallized at 293 K.

#### 2.2.2. Setting up IMISX plates, snap-cooling and storing wells   

The plates used for IMISXcryo are identical to those used for IMISX data collection (Huang *et al.*, 2015 ▸). These double-sandwich plates and how they are assembled and filled with protein-laden mesophase and precipitant solution have been described in detail separately (Supplementary Movie S1). Their use in IMISXcryo measurements requires a number of additional steps as outlined below.

**Figure 1:**
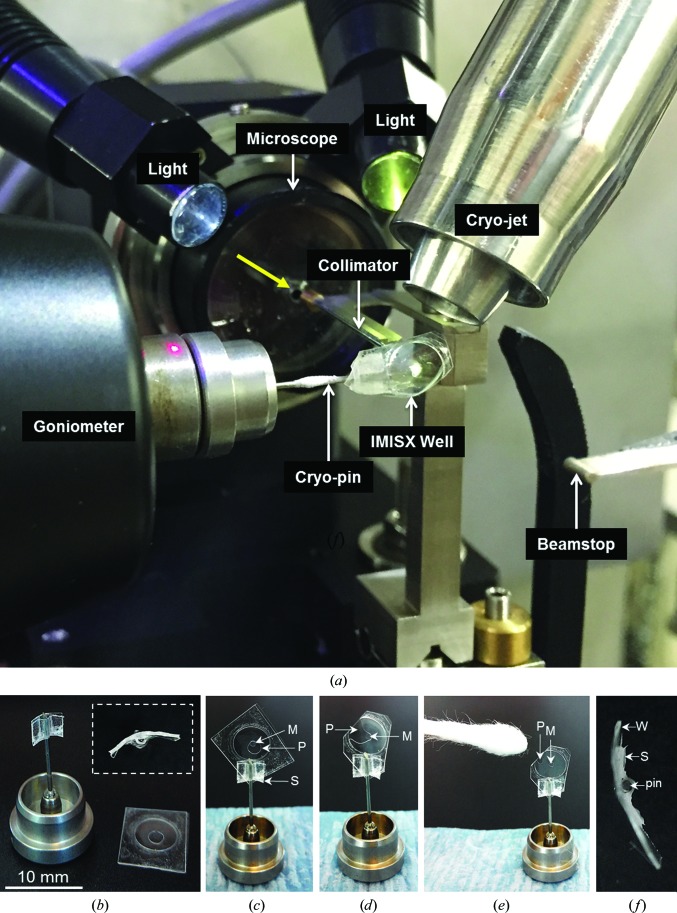
Experimental setup for IMISXcryo data collection at 100 K and the steps involved in sample preparation. (*a*) A view of an IMISX well on a goniometer with the crystal-laden mesophase bolus positioned in the X-­ray beam and in the cryostream at 100 K for SX data collection on beamline PXI (X06SA) at the SLS. The yellow arrow indicates the direction of the X-ray beam. (*b*) Goniometer base with composite support on the pin (left) ready for attachment of the IMISX well (bottom right). The insert (dashed square) is a view from above of the goniometer base (left) to show the slight curvature in the attached composite support. (*c*) IMISX well mounted on a goniometer base and seated on a moist paper towel. M, mesophase bolus with crystals; P, precipitant solution; S, composite support. (*d*) As in (*c*) where the well has been trimmed to fit into a cryo-vial and to provide access to the precipitant solution. (*e*) A cotton bud is used to wick away most of the precipitant solution from around the mesophase bolus. A small amount of precipitant is left in place to prevent callapse of the well. The mounted sample is snap-cooled in liquid nitrogen and placed in a cryo-vial for transfer to a Dewar. (*f*) A view of the well (W) and support (S) in (*e*) from above to show the slight curvature in the well that confers structural stability to the sample in the cryostream.

**Figure 2:**
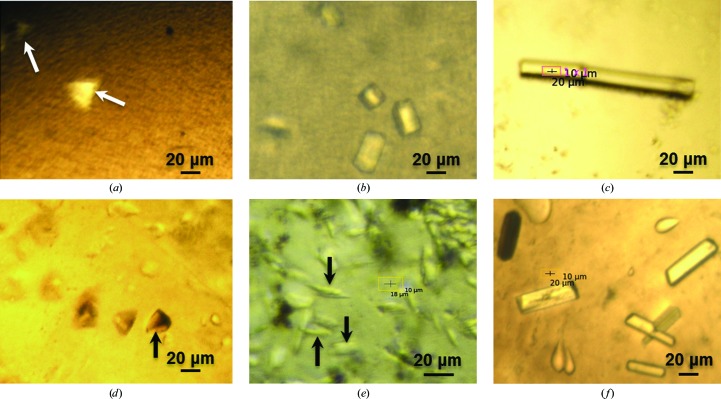
Images of crystals in IMISXcryo wells in a cryostream at 100 K as viewed by a high-resolution in-line microscope. (*a*) Insulin. (*b*) Lysozyme. (*c*) AlgE. (*d*) PepT_St_. (*e*) β_2_AR. (*f*) DgkA. Arrows point to crystals in the mesophase. DgkA crystals were grown at 277 K. All others were grown at 293 K.

*Step 1*. Place the filled, double-sandwich IMISX plate in a temperature-regulated chamber at 277 K or an incubator/imager (Rock Imager RI1500, Formulatrix, Waltham, Massachusetts, USA) at 293 K. Monitor for crystal growth by periodic inspection using bright-field and cross-polarized light microscopy. Record digital images of the mesophase bolus and the crystals therein to facilitate crystal identification for IMISXcryo at the beamline.
*Step 2*. Prepare a goniometer base modified to support the IMISX well for storage and data collection at cryogenic temperatures by assembling the following items: a goniometer base (SPINE standard) with an 18 mm SPINE standard pin, a 5 × 20 mm section of 50 µm thick COC film, two 5 × 2 mm sections of double-stick tape, a pointed object such as a glass-cutting tool or a sharp needle or pin, forceps and scissors (Supplementary Fig. S1 and Supplementary Movie S2). Remove the protective cover from one side of a piece of double-stick tape. Affix it, sticky side down, to the bottom of the COC film with the 5 mm edges aligned. With the glass cutter, needle or pin, make a small hole in the middle of the COC film just above the upper edge of the double-stick tape. Insert the pin of the goniometer base into the hole to the extent of 20 mm from the side without double-stick tape. Remove the second protective cover from the double-stick tape, fold the COC film up and around the pin and press firmly to tightly bond the two pieces of COC film together and to secure the pin between them. With thumb and forefinger, bend the COC film slightly (10–20°; Fig. 1 ▸ *b*) around the long axis of the goniometer pin with the concave surface towards the short COC flap. Remove the protective cover from the second piece of double-stick tape and affix it to the bottom of the COC film on its long flap. With sharp scissors, cut away the long, untapped COC film and trim the remaining support symmetrically on either side of the pin to an overall width of 2 mm. Gently repeat the bending process to reinforce the slight curvature in the composite COC-tape support. This gives a 2 × 2 mm square and slightly curved support to which the excised well can be attached (Fig. 1 ▸ *b*). Step 2 takes about 3 min to complete.
*Step 3*. With a glass-cutting tool and a straight edge, score lines in the No. 1.5 cover glass of the double-sandwich plate around the well of interest and remove the cover glass. Use a sharp blade or scalpel to free a square-shaped section of the COC sandwich plate about the exposed well and retrieve it with forceps. Reseal the opened plate with the cover glass and tape and store it in a sealed, humid chamber until required. Step 3 takes about 1 min to complete (Supplementary Movie S3).
*Step 4*. Remove the protective cover from the double-stick tape on the square composite support on the goniometer base prepared in Step 2. Affix the excised well to the support with the diagonal axis of the well aligned with the long axis of the pin for maximum contact between the two. Use forceps to press the well and the support firmly together to ensure a strong bond (Figs. 1 ▸ *b* and 1 ▸ *c*; Supplementary Movie S3). The well should have acquired a slight curvature imposed by the curvature in the support (Fig. 1 ▸ *f*). Set the pin/goniometer base aside on a moist tissue for subsequent manipulations. The orientation of the ‘flat’ face of the well should be recorded on the bottom of the goniometer base (a marker works well) to facilitate proper robotic and manual positioning of the sample in the X-ray beam and in the cryostream. With the base on a magnetic wand for easy handling, trim the corners of the well with sharp scissors, leaving a continuous strip of double-stick tape around the edge of the well for structural stability (Fig. 1 ▸ *d*). The trimmed well must be small enough to fit comfortably into a cryo-vial. Trim a minimum off one side of the well to expose the precipitant solution (Fig. 1 ▸ *d*). By touching the precipitant with tissue paper or a cotton bud, gently wick away most of the precipitant from around the mesophase bolus (Fig. 1 ▸ *e*). A small amount of precipitant left in the well, as shown in Fig. 1 ▸(*e*), helps to prevent collapse of the well windows and a thinning of the mesophase which can damage crystals. Immediately, and with maximum speed, plunge the well into a loading Dewar filled to the brim with liquid nitrogen (Warkentin *et al.*, 2006 ▸). When the sample has equilibrated thermally, transfer it into a precooled storage puck (containing empty cryo-vials) in the same loading Dewar. Using the mark on the bottom of the goniometer base, orient the base in the puck to ensure that the well will be aligned correctly in the beam and in the cryostream upon robotic mounting. Place the puck in a shipping Dewar and ship to the synchrotron. Step 4 takes about 3 min to reach the point where the sample has been secured in a cryo-vial in a puck.
*Step 5*. At the beamline, samples can be mounted on the goniometer manually or using a robot (Supplementary Movie S4). In either case, care should be taken to ensure that the sample is mounted with the face of the well normal to the beam. Check the orientation of the well with respect to the cryostream and make adjustments as needed to ensure that the well edge faces into the stream, where both sides of the well are cooled equally and the mesophase bolus is in the centre of the stream. Position the bolus in the cross-hairs of the high-resolution in-line sample-viewing microscope which corresponds to the position of the beam (Fig. 1 ▸ *a*). (For reference, we define the *x*, *y* and *z* positioning of the crystal as follows. *x* corresponds to the crystal position along the rotation axis of the goniometer which is aligned to intersect orthogonally with the X-ray beam axis. *z* corresponds to the position along the beam axis and *y* to the position along the axis perpendicular to both *x* and *z*.) The in-line microscope has a continuously variable magnification from 2.5-fold to 30-fold. The COC sandwich plate is optically transparent and crystals are usually visible when viewed *in situ* with the microscope (Fig. 2 ▸). Adjust the *x*, *y* and *z* coordinates of a chosen crystal in the well by means of the motorized gonio­meter to position the crystal in the focal plane of the microscope that includes the rotation axis of the goniometer and the cross-hairs of the camera/microscope. This ensures that the crystal is in position for data collection when only a few degrees of rotation are needed. In those cases where a large rotation range is possible, data can be collected in wedges and adjustment in the *x*, *y* and *z* positions for each wedge may be needed to keep the crystal in the beam and on the rotation axis. The SLS data-acquisition software (*DA*+) was used for semi-automated, high-throughput crystal picking. Crystal positions are recorded for use in subsequent automated, sequential SX data collection. For large crystals, multiple positions on the same crystal can be selected, taking care to provide a sufficient distance between them to avoid spillover of radiation damage. Data are collected iteratively with due regard to beam and crystal characteristics. Repeat the ‘select/pick and shoot’ protocol within a well and extend to additional wells, as needed, until data of sufficient quality and completeness have been collected. The software recognizes which crystals have been X-rayed and avoids re-using them. These are flagged on the computer-screen image from the on-axis microscope and are colour-coded by number of reflections detected at that data-collection site in the well. Occasionally, the snap-cooling process causes the mesophase to turn opaque such that the crystals are no longer visible in the IMISX well. In this case, diffraction raster scanning is used to locate and to centre crystals. Step 5 takes at most 1 min to reach the crystal-picking stage.

#### 2.2.3. Data collection: IMISXcryo and IMISX at RT   

X-ray diffraction experiments were carried out on protein crystallography beamlines PX I (X06SA) and PX II (X10SA) at the Swiss Light Source, Villigen, Switzerland. With the exception of a reference data set for insulin recorded at RT, all data were collected at 100 K with IMISX wells in a cryostream. Measurements were made in steps of 0.1–0.4° at speeds of 0.33–4 deg s^−1^ with a PILATUS 6M-F detector operated in a continuous/shutterless data-collection mode at a frame rate of 3.3–20 Hz and a sample-to-detector distance of between 20 and 60 cm. Native data from insulin, PepT_St_, β_2_AR and DgkA crystals were measured with a 10 × 18 µm X-ray microbeam at wavelengths and flux values of 0.97857 Å and 5.9 × 10^10^ photons s^−1^, 0.97852 Å and 7.4 × 10^10^ photons s^−1^, 1.0332 Å and 3.2 × 10^11^ photons s^−1^ and 1 Å and 1.8 × 10^11^ photons s^−1^, respectively, on beamline PX II. For native lysozyme data collection, a 10 × 30 µm X-ray microbeam with a flux of 8.0 × 10^10^ photons s^−1^ at 1 Å was used on beamline PX II. A 15 × 20 µm beam with a flux of 5.0 × 10^11^ photons s^−1^ at 0.97856 Å was used for native AlgE data collection on beamline PX I. A 15 × 20 µm X-ray microbeam with a wavelength of 2.0664 Å delivering 4.3 × 10^10^ photons s^−1^ on beamline PX I was used for insulin sulfur SAD phasing. For bromine SAD phasing, diffraction data were collected at the absorption edge of 0.91881 Å on beamline PX II. The X-ray beam size and flux values were 10 × 30 µm and 3.4 × 10^10^ photons s^−1^, respectively. Room-temperature insulin data were measured with a 10 × 30 µm X-ray beam delivering 2.0 × 10^11^ photons s^−1^ at 1.0 Å on beamline PX II.

#### 2.2.4. Data collection: harvested at 100 K   

For reference, data were collected from crystals that had been grown *in meso* and that were harvested and snap-cooled in liquid nitrogen by conventional methods. For this purpose, the COC plates were opened with a scalpel to expose the mesophase. A 20–50 µm cryoloop was used to retrieve the crystal or crystals from the bolus, with as little adhering mesophase as possible, and they were immediately snap-cooled in liquid nitrogen without added cryoprotectant. The loops were stored in Dewars and shipped to the SLS for data collection. Insulin, PepT_St_ and β_2_AR crystals were measured with a 10 × 18 µm X-ray beam with a wavelength of 1.0332 Å and flux values of 6.0 × 10^10^, 6.0 × 10^10^, 9.0 × 10^10^ photons s^−1^ on beamline PX II, respectively. DgkA reference data were collected with a smaller X-ray beam measuring 5 × 10 µm^2^ and delivering 3.2 × 10^10^ photons s^−1^ on beamline PX I.

#### 2.2.5. Radiation damage   

Radiation damage in macromolecule diffraction experiments at 100 K has been studied extensively (Ravelli & Garman, 2006 ▸). For native data collection, the accumulated dose limit is about 20–30 MGy (Henderson, 1990 ▸; Owen *et al.*, 2006 ▸; Holton, 2009 ▸; Liebschner *et al.*, 2015 ▸). In the case of experimental phasing using native or incorporated heavy atoms, the dose limit is much reduced owing to specific radiation damage and the need for data of high quality. Accordingly, the exact dose limit depends on the number and identities of the heavy atom(s) and their chemical environment. Generally, crystals should not be exposed to more than a few megagrays. The accumulated dose per crystal registered in the current study was calculated (Holton, 2009 ▸) and the values are included in Table 1 ▸. These estimated values are accurate to within a factor of two at best and are overestimates when the crystal is larger than the X-ray beam footprint and a relatively wide rotation range is used for data collection. With these considerations in mind, all IMISXcryo data were collected within the aforementioned limits.

#### 2.2.6. Data processing and merging   

All ‘conventional cryo-data’ collected from harvested crystals in loops at 100 K were processed with *XDS* and scaled with *XSCALE*.

The IMISXcryo diffraction data were processed and scaled using *XDS* and *XSCALE* as described previously by Huang *et al.* (2015 ▸). Briefly, each data set was indexed and integrated with *XDS*. The selection of data sets was carried out in two steps. Firstly, all data sets were scaled together using *XSCALE* with the ‘MINIMUM_I/SIGMA=0.0’ option. Data sets with ISa values below a certain threshold were rejected (Diederichs, 2010 ▸). Secondly, the correlation coefficient of each scaled individual data set and of the merged data set minus that data set (CC_set_) was calculated. Data sets with CC_set_ below a certain threshold were excluded. Threshold values for ISa and CC_set_ are given in Table 2 ▸. All steps were carried out in an iterative manner. The second step was executed using a custom script. The final data sets were scaled and merged with *XSCALE*. Data-collection and processing statistics are provided in Tables 2 ▸ and 3 ▸.

#### 2.2.7. Structure determination and refinement   

Molecular replacement (MR) was used to search for a solution in the native insulin, lysozyme, AlgE, PepT_St_, β_2_AR and DgkA data sets with *Phaser* (McCoy *et al.*, 2007 ▸) using PDB entries 9ins (Gursky *et al.*, 1992 ▸), 3tmu (Kmetko *et al.*, 2011 ▸), 4afk (Tan *et al.*, 2014 ▸), 4d2c (Lyons *et al.*, 2014 ▸), 2rh1 (Cherezov *et al.*, 2007 ▸) and 3ze3 (Li *et al.*, 2013 ▸), respectively, as model templates. The SAD method was employed for experimental phasing using anomalous diffraction data sets from native insulin (insulin-S) and bromine-derivatized lysozyme (lyso-Br) crystals. Heavy-atom location, structure phasing and density modification were performed with the *HKL*2*MAP* interface to *SHELXC*, *SHELXD* and *SHELXE* (Sheldrick, 2010 ▸) for insulin-S and lyso-Br. Heavy-atom substructures of insulin-S and lyso-Br were identified with 1000 and 100 *SHELXD* trials, respectively, and initial phasing employed 20 cycles of *SHELXE* density modification with autobuilding of the protein backbone trace. All models were completed with manual inspection using *Coot* (Emsley *et al.*, 2010 ▸). *PHENIX* (Adams *et al.*, 2010 ▸) and *BUSTER* (Bricogne, 1993 ▸; Roversi *et al.*, 2000 ▸) were used during the refinement of all structures, with the ‘strategy’ options of ‘individual sites’, ‘real space’, ‘individual atomic displacement parameter’, ‘ordered solvent’ and ‘target weight optimization’ turned on. Refinement statistics are reported in Tables 2 ▸ and 3 ▸. Figures were generated with *PyMOL* (http://www.pymol.org).

All diffraction data and refined models have been deposited in the Protein Data Bank as entries 5d52, 5d53, 5d54, 5d56, 5d57, 5d58, 5d59, 5d5a, 5d5b, 5d5c, 5d5d, 5d5e and 5d5f.

## 3. Results   

The success of the IMISXcryo approach to high-throughput structure determination for crystals grown by the *in meso* method without the need for the time-consuming and inefficient crystal-harvesting step is illustrated in this section. We begin by demonstrating that the method works with the test soluble proteins insulin and lysozyme. In the case of insulin, structures were solved by MR and by sulfur SAD phasing. For lysozyme, both MR and bromine SAD were used. The integral membrane proteins in the study included AlgE, PepT_St_, β_2_AR and DgkA, and MR phasing was used with all four. The point of this paper is not to describe the structures but rather to demonstrate that the IMISXcryo method works easily and efficiently with a range of protein types and that it can be used both for high-resolution native data collection and for experimental phasing.

### 3.1. Native insulin: molecular-replacement phasing   

Porcine pancreas insulin was selected as a model test protein with which to explore the feasibility of the IMISXcryo approach. Despite the fact that it is a soluble protein, like lysozyme it crystallizes quickly and reproducibly using the *in meso* method (Aherne *et al.*, 2012 ▸) and the crystals, whilst small, are usually of very high diffraction quality. It crystallizes in space group *I*2_1_3, with unit-cell parameter 78.4 Å. Insulin has a high content of sulfur-containing amino acids. Accordingly, it is an ideal test protein with which to perform sulfur SAD phasing (§3.3).

Crystals of insulin grew in IMISX plates within 48 h and were stable for days at 293 K. The crystals typically ranged from 20 to 30 µm in maximum dimension (Fig. 2 ▸ *a*). As a prelude to IMISXcryo measurements, a control data set was collected in IMISX mode at 293 K. Measurements were made on 25 crystals in two wells with a 10 × 30 µm beam at 1.0 Å delivering 2.0 × 10^11^ photons s^−1^ at 0.2° and 0.05 s per step (Table 1 ▸). 5° of data were collected from each crystal, providing a total useful angular range of 125° (Table 4 ▸). The structure was solved by MR with PDB entry 9ins as a search model and was refined to a resolution of 1.8 Å with an *R*_work_ and *R*_free_ of 0.17 and 0.21, respectively (Table 2 ▸). In the highest resolution shell, the completeness was 100%, CC_1/2_ was 0.46 and 〈*I*/σ(*I*)〉 was 0.88. The 100% completeness is consistent with the crystals being randomly oriented in the IMISX wells.

We next proceeded to the IMISXcryo measurements with crystals, as described above, suspended in a snap-cooled bolus in a COC sandwich well at 100 K. Despite their small size and the cryo-treatment, the crystals were readily visible using the in-line microscope (Fig. 2 ▸ *a*). In this case, a complete data set to 1.5 Å resolution was collected from a single 30 × 30 × 30 µm crystal. The X-ray beam employed had a wavelength of 0.97857 Å, dimensions of 10 × 18 µm and delivered a flux of 5.9 × 10^10^ photons s^−1^. Data were collected over a 60° range in 0.4° steps at 0.2 s per step (Tables 1 ▸ and 4 ▸). The structure was solved by MR, as above, and refined to a resolution of 1.5 Å with an *R*_work_ and *R*_free_ of 0.16 and 0.17, respectively (Table 2 ▸). The highest resolution shell had a completeness of 100%, a CC_1/2_ value of 0.46 and an 〈*I*/σ(*I*)〉 of 1.21.

For reference, a crystal was harvested from the same or similar plates to those used for IMISX and IMISXcryo with a cryoloop, snap-cooled in liquid nitrogen and used for data collection at 100 K. Phasing was performed by MR, as above. Data-collection and refinement statistics for this single snap-cooled crystal are presented in Table 3 ▸. The crystal diffracted to 1.5 Å resolution with a completeness of 100%, a CC_1/2_ of 0.55 and an 〈*I*/σ(*I*)〉 of 1.49 in the highest resolution shell. The structure was refined with an *R*_work_ and *R*_free_ of 0.18 and 0.19, respectively (Table 3 ▸).

The electron-density maps for insulin obtained by the IMISX and IMISXcryo data-collection modes are of high quality and the corresponding models are virtually identical [backbone root-mean-square deviation (r.m.s.d.) of 0.138 Å over 51 residues] to that obtained from the reference loop-harvested crystals at 100 K. The data-quality and refinement statistics are comparable (Tables 2 ▸ and 3 ▸). The maps reveal the presence of two phosphate ions and a molecule of PEG 400 in both the IMISXcryo and reference loop-harvested samples (Supplementary Table S1). These results demonstrate convincingly that the IMISXcryo method works remarkably well with insulin crystals and, importantly, without the need for crystal harvesting. For the purposes of IMISXcryo data collection, a single bolus of mesophase was used corresponding to 200 nl mesophase, 120 nl lipid and 2.8 µg protein (Table 4 ▸).

### 3.2. Native lysozyme: molecular-replacement phasing   

Lysozyme was used as the second model soluble protein with which to evaluate the IMISXcryo method. Crystals grew in IMISX plates within 1 h and were stable in the mesophase for at least 2 d at 293 K (Fig. 2 ▸ *b*). Using the IMISXcryo method, two crystals measuring 15 × 15 × 30 µm provided a complete data set to 1.7 Å resolution. The X-ray beam employed had a wavelength of 1.0 Å, measured 10 × 30 µm and delivered a flux of 8 × 10^10^ photons s^−1^ (Table 1 ▸). Data were collected over a 50° range in 0.1° steps at 0.1 s per step, and 40° of data from each crystal, representing 80° of merged data, were used in data processing (Tables 1 ▸ and 4 ▸). The structure was solved by MR with PDB entry 3tmu as a model and was refined to a resolution of 1.7 Å with an *R*_work_ and *R*_free_ of 0.18 and 0.22, respectively (Table 2 ▸). The highest resolution shell had a completeness of 100%, a CC_1/2_ value of 0.32 and an 〈*I*/σ(*I*)〉 of 1.10. These IMISXcryo data compare very favourably with those obtained using loop-harvested crystals at 100 K. The structures are almost identical, with an r.m.s.d. of 0.351 Å over 129 residues (Supplementary Table S1). For the purposes of IMISXcryo data collection, a single bolus of mesophase was used corresponding to 200 nl mesophase, 120 nl lipid and 4 µg protein (Table 4 ▸).

For comparison, the IMISX structure determined at RT had a resolution of 1.8 Å. Measurements were made with a 10 × 18 µm beam at 1.0 Å delivering 3.0 × 10^11^ photons s^−1^ at 0.2° and 0.05 s per step. In this case. however, 113 crystals from two wells were required for a complete data set, with each crystal providing 1.2° of data. This dramatic difference in the number of crystals required for structure determination highlights an advantage of the IMISXcryo approach.

### 3.3. Native insulin: sulfur SAD phasing   

The IMISXcryo method works well for phasing by MR. A more stringent test of diffraction data quality is to determine whether the method can be used for sulfur SAD phasing. To this end, 60° of data were collected from each of fourteen 30 × 30 × 30 µm insulin crystals in a single well and six crystals were used in the final merging, corresponding to a total of 360° of data. Measurements were made with a 15 × 20 µm beam at 2.06643 Å and a flux of 4.3 ×10^10^ photons s^−1^ in 0.1° steps at 0.1 s per step (Table 1 ▸). In the highest resolution shell, the completeness was 99.5%, the CC_1/2_ was 0.86 and 〈*I*/σ(*I*)〉 was 2 (Table 2 ▸). The measurable anomalous signal extended to 2.4 Å resolution. The structure was solved by sulfur SAD phasing with CC_all_ and CC_weak_ of 28.5 and 19.0, respectively, in *SHELXD* using the ‘resolving disulfide’ option and with well separated contrast between correct and inverted hands in *SHELXE* (Supplementary Figs. S2*a* and S2*b*). *SHELXE* with auto-tracing built a main-chain model with 27 out of a total of 51 residues. The final model was refined to a resolution of 2.4 Å with an *R*_work_ and *R*_free_ of 0.17 and 0.22, respectively (Table 2 ▸). The electron-density maps and the models obtained for insulin structures solved by MR and by sulfur SAD are very similar, with a backbone r.m.s.d. value of 0.134 Å over 51 residues. The anomalous difference map, contoured at 5σ, shows three well defined lobes of density attributed to three super-sulfurs (disulfides) in the IMISXcryo data (Supplementary Fig. S3*a*). For the purposes of obtaining this sulfur SAD structure by the IMISXcryo method, 200 nl of mesophase representing 2.8 µg protein and 120 nl 9.9 MAG were used (Table 4 ▸). These results show that sulfur SAD phasing is possible by the IMISXcryo method as applied to insulin crystals. The quality of the data attainable by the method is therefore very high.

### 3.4. Lysozyme: bromine SAD phasing   

Having demonstrated that sulfur SAD phasing was possible by the IMISXcryo method with insulin crystals, there was little doubt that bromine SAD phasing would work with lysozyme crystals grown in the presence of NaBr. However, this was carried out to further test the method and to compare the results with the data obtained previously from harvested, *in meso*-grown crystals at 100 K. The brominated lysozyme crystals grown in IMISX plates were relatively large at 15 × 15 × 30 µm (Fig. 2 ▸ *b*). The beam size was adjusted to 10 × 30 µm to optimize the signal to noise. Data were collected at 0.91881 Å and with a flux of 3.4 × 10^10^ photons s^−1^ from eight crystals in a single well. Each crystal provided 30° of data recorded in steps of 0.1° at 0.1 s per step (Table 1 ▸). The final merged data reached 1.5 Å resolution and the anomalous signal extended to 2.0 Å resolution. The completeness was 92%, CC_1/2_ was 0.38 and 〈*I*/σ(*I*)〉 was 0.97 in the highest resolution shell (Table 2 ▸). The structure was phased easily with *SHELXC*/*D*/*E* via the *HKL*2*MAP* interface. The CC_all_ and CC_weak_ values were 26.9 and 15.8, respectively, in *SHELXD* and the correct handedness was clearly identified in *SHELXE* (Supplementary Figs. S2*c* and S2*d*). The anomalous difference map contoured at 5σ shows well defined lobes of density attributed to five bromide ions in the IMISXcryo data (Supplementary Fig. S3*b*). The structure was refined to a resolution of 1.5 Å with an *R*_work_ and *R*_free_ of 0.18 and 0.21, respectively (Table 2 ▸). These IMISXcryo data compare very favourably with those obtained using loop-harvested crystals at 100 K. The structures are almost identical, with an r.m.s.d. value of 0.098 Å over 129 residues (Supplementary Table S1). The advantage of the IMISXcryo method over the IMISX method, in which data are collected at ambient temperature, is apparent in the number of crystals that are required for phasing. The former method used eight crystals, while the latter required 239. This bromine SAD phasing demonstration by the IMISXcryo method consumed 200 nl mesophase representing 4 µg protein and 120 nl 9.9 MAG (Table 4 ▸).

The anomalous signal from bromine is similar to that from selenium. Thus, while selenium-labelled protein was not used in this study, the results obtained with bromine SAD suggest that selenium SAD phasing should be possible using IMISXcryo. This has been confirmed in a separate study employing a membrane protein (unpublished work).

### 3.5. Native AlgE: molecular-replacement phasing   

The *in meso* method was introduced for use with membrane proteins. Thus far, we have shown that the IMISXcryo method for data collection works well with soluble proteins. The next step was to evaluate its usefulness with membrane proteins. The first protein used for this purpose was AlgE, an alginate transporter that resides in the outer membrane of *P. aeruginosa* (Tan *et al.*, 2014 ▸). In IMISX plates, crystals of this 18-stranded β-barrel protein grew to dimensions of up to 15 × 15 × 150 µm (Fig. 2 ▸ *c*). Data were collected in steps of 0.1° and 0.1 s per step with a beam measuring 15 × 20 µm at 0.97856 Å and a flux of 5.0 × 10^11^ photons s^−1^ (Table 1 ▸). Because the crystal used for data collection was long (150 µm), it was possible to collect all of the required data from four separate locations on the one crystal in the IMISX well. 20–30° of data were combined from the four sites to give a total of 100° of data. The structure was solved by MR using PDB entry 4afk as a model. Data-collection and refinement statistics for this IMISXcryo crystal are presented in Table 2 ▸. The crystals, which belonged to space group *P*2_1_2_1_2_1_, diffracted to 2.4 Å resolution with a completeness of 90.8%, a CC_1/2_ of 0.62 and an 〈*I*/σ(*I*)〉 of 1.45 in the highest resolution shell. The structure was refined with an *R*_work_ and *R*_free_ of 0.22 and 0.26, respectively (Table 2 ▸). The corresponding electron-density map was of high quality and the model was similar (backbone r.m.s.d. of 0.599 Å over 403 residues) to that obtained using crystals harvested from IMISX plates with data collected at 100 K, where the resolution was 2.9 Å (Supplementary Table S1). The data-quality and refinement statistics were comparable between IMISXcryo data and the previously published IMISX data obtained from 175 crystals at RT. The r.m.s.d. of backbone atoms was 0.799 Å over 403 residues. The data used to generate the IMISXcryo structure were obtained with 50 nl of mesophase representing 0.25 µg protein and 25 nl 7.8 MAG (Table 4).

**Figure 3:**
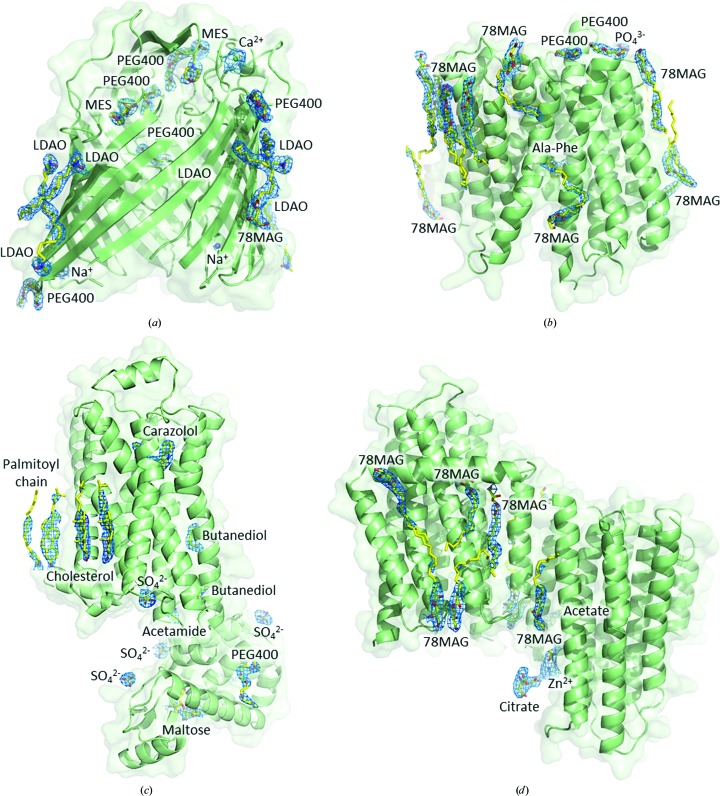
Structures of four integral membrane proteins solved using the IMISXcryo method. (*a*) AlgE. (*b*) PepT_St_. (*c*) β_2_AR. (*d*) DgkA. 2*F*_o_ − *F*_c_ electron-density maps for ligands, lipids, ions and other nonproteinaceous molecules in the structures are shown as a blue mesh contoured at 1σ. Structures are shown in cartoon and surface representation and are coloured light green. Ions are shown as spheres: calcium, green; sodium, purple; zinc, grey. The resolutions of the corresponding structures are 2.4 Å (AlgE, PepT_St_), 2.5 Å (β_2_AR) and 2.8 Å (DgkA). Stick models show C (yellow), N (blue) and O (red) atoms.

Dimannuronate, a surrogate for the alginate polysaccharide transported by AlgE (Tan *et al.*, 2014 ▸), was included with the protein during crystallogenesis. The expectation was that it would bind in the electrostatic pore midway across the β-barrel through which alginate is proposed to pass en route to the extracellular space. Convincing density for this small molecule was not obvious in the corresponding electron-density map (Fig. 3 ▸ *a*). However, density for two MES buffer molecules was clearly visible.

### 3.6. Native PepT_St_: molecular-replacement phasing   

The second membrane protein used to test the IMISXcryo method was the α-helical peptide transporter PepT_St_. Pyramid-shaped crystals belonging to space group *C*222_1_ grew readily in the IMISX plates to dimensions of 20 × 20 × 30 µm and were clearly visible using the in-line microscope (Fig. 2 ▸ *d*). As reported previously, the radiation-sensitivity and weakly diffracting nature of PepT_St_ crystals only allowed about 0.6° of data to be collected from each small crystal with an attenuated X-ray beam at RT. As a result, 572 crystals were required to provide a complete data set to 2.8 Å resolution. Indeed, radiation damage may have also limited the achievable diffraction resolution. By contrast, using the IMISXcryo method, 60–90° of data could be collected from a single crystal with a 10 × 18 µm beam delivering 7.4 × 10^10^ photons s^−1^ and a wavelength of 0.97852 Å (Table 1 ▸). A complete data set was obtained by merging a total of 120° of data from just two crystals in a single well. The diffraction resolution was 2.4 Å, with a completeness of 98.8%, a CC_1/2_ value of 0.62 and an 〈*I*/σ(*I*)〉 of 2.18 in the highest resolution shell (Table 2 ▸). The structure was solved by molecular replacement using PDB entry 4d2c as a search model and refined to an *R*_free_ and *R*_work_ of 0.21 and 0.25, respectively. For reference, diffraction data were also collected from a single crystal harvested directly from an IMISX plate (Table 3 ▸). The data-quality and refinement statistics are similar between the IMISXcryo data, the loop-harvested data and the previously published IMISX data. The diffraction resolution is significantly higher for the IMISXcryo data (2.4 Å) than for the IMISX RT data (2.8 Å). The r.m.s.d. of backbone atoms is 0.158 Å over 448 residues between the IMISXcryo structure and the loop-harvested structure (Supplementary Table S1). For IMISXcryo data collection, 50 nl of mesophase representing 25 nl 7.8 MAG and 0.25 µg protein were used (Table 4 ▸).

**Figure 4:**
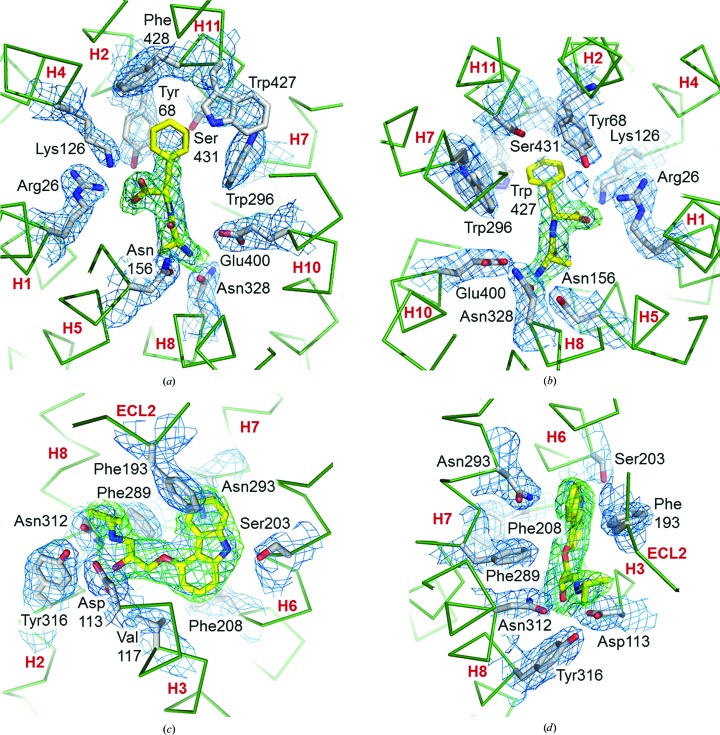
Electron-density maps of ligands bound to PepT_St_ and β_2_AR in crystal structures solved by the IMISXcryo method at resolutions of 2.4 and 2.5 Å, respectively. (*a*, *b*) Views into the peptide-binding pocket of PepT_St_ reveal the dipeptide Ala-Phe in well defined density. (*c*, *d*) Views into the ligand-binding pocket of β_2_AR reveal the partial inverse agonist carazolol in well defined density. Ligands are shown with yellow C atoms and with an *F*_o_ − *F*_c_ map (green mesh) contoured at 3σ. 2*F*_o_ − *F*_c_ maps are contoured at 1σ (blue mesh). Relevant residues are highlighted as sticks with grey C atoms. The protein backbone is shown in thin ribbon representation coloured green. Helices (H) and an extracellular loop (ECL) are indicated following the notation in the original literature. Stick models include N (blue) and O (red) atoms.

The IMISXcryo crystallization trials on PepT_St_ were conducted in the presence of the dipeptide Ala-Phe, which is a known substrate of this transporter (Lyons *et al.*, 2014 ▸). Convincing density for Ala-Phe in the canonical peptide-binding pocket of the transporter was visible in the electron-density map (Figs. 4 ▸ *a* and 4 ▸ *b*). Indeed, electron density was seen for most of the phenyl ring of the C-terminal residue, only part of which was visible in the original published structure (Lyons *et al.*, 2014 ▸). The IMISXcryo structure also includes more bound MAG lipids (Fig. 3 ▸ *b*).

### 3.7. Native β_2_AR: molecular-replacement phasing   

The β_2_AR used in this study is the carazolol-bound form of a fusion between the human β_2_-adrenoreceptor and T4 lysozyme (Rosenbaum *et al.*, 2007 ▸). This was the first non-rhodopsin GPCR to have its structure solved crystallo­graphically using crystals grown by the *in meso* method. Carazolol is a tightly bound partial inverse agonist with a low off-rate that stabilizes the receptor in a particular conformation. The original structure was solved and refined to a resolution of 2.4 Å using a total of 27 loop-harvested microcrystals (average dimensions 5 × 15 × 30 µm) at 100 K. The reason for including β_2_AR in the current study was to investigate the applicability of the IMISXcryo method to GPCRs, in which there is much interest, reflecting their physiological and medical importance.

Crystals of β_2_AR grew in IMISX plates at 293 K in 1–2 d. The crystals were cigar-shaped with maximum dimensions of 5 × 10 × 30 µm (Fig. 2 ▸ *e*). The space group was *C*2, with unit-cell parameters *a* = 108.04, *b* = 170.58, *c* = 40.44 Å, α = 90, β = 106.30, γ = 90°. Earlier attempts at RT structure determination by the IMISX method were not successful, with the highest resolution reflections reaching 4 Å at best. The low-symmetry monoclinic space group, which requires a large angular coverage of reciprocal space and much better diffracting crystals, worked against complete data-set collection by the IMISX RT method. Reference data were collected at 100 K from β_2_AR crystals loop-harvested from the IMISX plate. In our hands, these diffracted and provided structures to no better than 3.5 Å resolution (Table 3 ▸). Using the IMISXcryo method, however, we were able to collect the requisite data to solve a structure at 2.5 Å resolution (Table 2 ▸). This was achieved using a beam measuring 10 × 18 µm delivering 3.2 × 10^11^ photons s^−1^ at 1.0332 Å wavelength. The data were collected with 0.1° and 0.3 s per step from 149 crystals in one well with 3° of data per crystal. 104 partial data sets were processed satisfactorily and merged, providing a final data set with a total angular coverage of 312° (Table 4 ▸). The highest resolution shell had a completeness of 91.0%, a CC_1/2_ value of 0.21 and an 〈*I*/σ(*I*)〉 of 1.11 (Table 2 ▸). The structure was solved by MR using PDB entry 2rh1 as a search model and refined to *R*_free_ and *R*_work_ values of 0.21 and 0.26, respectively. The carazolol ligand, covalently linked fatty acid and bound cholesterol are well defined in the electron-density map (Figs. 3 ▸ *c*, 4 ▸ *c* and 4 ▸ *d*). As expected, the published (Cherezov *et al.*, 2007 ▸) and the IMISXcryo structures are remarkably similar, with a backbone r.m.s.d. value of 0.200 Å over 442 residues (Supplementary Table S1). The IMISXcryo data were collected using 30 nl of mesophase representing 0.58 µg protein and 18 nl 9.9 MAG/cholesterol mixture (Table 4 ▸).

### 3.8. Native DgkA: molecular-replacement phasing   

DgkA is a kinase that resides in the inner membrane of *E. coli*. It is responsible for the ATP-dependent synthesis of phosphatidic acid from diacylglycerol (Li *et al.*, 2013 ▸, 2015 ▸). Crystals of this protein grow optimally in 7.8 MAG at 277 K. DgkA is therefore an example of a protein that produces crystals at a temperature other than ambient, where *in situ* data collection, as implemented in the IMISX method, cannot be performed conveniently at most synchrotron MX beamlines. Increasingly, we find that membrane-protein targets crystallize optimally by the *in meso* method at 277 K. The IMISXcryo method is ideal for such targets because the crystals can be grown at 277 K and then snap-cooled and stored in liquid nitrogen for shipping in Dewars to a synchrotron for data collection at 100 K.

Crystals of DgkA measuring 20 × 20 × 50 µm grew within 4 d at 277 K in IMISX plates (Fig. 2 ▸ *f*). Reference measurements were made with a single crystal loop-harvested from the plates for snap-cooling in liquid nitrogen and for data collection at 100 K. IMISXcryo measurements were made using 12 crystals in five wells with a 10 × 18 µm beam at 1.0 Å delivering 1.8 × 10^11^ photons s^−1^ (Table 1 ▸). Data were collected with 0.1 s and 0.1° per step. Complete data from the 12 crystals came from a total of 280° of data gathered at 20–40° per crystal. The highest resolution shell had a completeness of 98.6%, a CC_1/2_ value of 0.36 and an 〈*I*/σ(*I*)〉 of 1.00 (Table 2 ▸). The structure was determined in space group *P*2_1_2_1_2_1_ to a resolution of 2.8 Å by MR phasing with PDB entry 3ze3 as a search model (Table 2 ▸; Fig. 3 ▸ *d*). The reference structure determined with a loop-harvested crystal was solved to a resolution of 2.8 Å (Table 3 ▸). The data-quality and refinement statistics are similar, as are the two structures, which have an r.m.s.d. value of 0.238 Å over 585 residues (Supplementary Table S1). These results show that the IMISXcryo method works with crystals that grow at temperatures other than ambient. The IMISXcryo measurements used 250 nl of mesophase representing 1.5 µg DgkA and 125 nl 7.8 MAG (Table 4 ▸).

## 4. Discussion   

### 4.1. An evaluation of the IMISXcryo method   

In the following, the pros and cons of the IMISXcryo method are considered.

#### 4.1.1. Delivering on design specifications   

With four integral membrane proteins and two soluble proteins, the results of this study highlight the features of the IMISXcryo method for *in situ* diffraction data collection from crystals grown *in meso* without the need for crystal harvesting. The high quality of the data forthcoming by this method was demonstrated by the fact that experimental phasing *via* sulfur and bromine SAD was realised. That direct harvesting of crystals from glass sandwich plates is not required makes the method very attractive, especially with small crystals in the more fluid sponge phase. With less mesophase and crystal handling, the expectation is that the structures obtained by the IMISXcryo method should be as good as or better than those obtained from loop-harvested crystals at 100 K. For the most part, the results reported here are consistent with this prediction.

The IMISXcryo method has delivered to design specifications. It provides the many advantages of the original IMISX method for performing *in situ* serial crystallography while avoiding or minimizing the problems and limitations associated with data collection at RT. As implemented, data collection with IMISXcryo wells can be performed using standard goniometer bases, pucks, Dewars, cryo-jets and wet-mounting crystal-handling robots (with vials). Dry-mounting robots should also work given that the gripper has enough space to accommodate the well. The method provides indefinite storage of samples in liquid nitrogen from any time point in the crystal-growth cycle and from any growth temperature. Since measurements are performed at 100 K there is a massive reduction in radiation damage and an extension to crystal lifetimes to give more useful data per crystal. It should be possible for groups performing *in meso* crystallization and with access to a modern synchrotron MX beamline to implement the IMISXcryo method with ease.

#### 4.1.2. The cryogenic advantage   

The IMISXcryo method uses wells with crystal-laden mesophase that have been snap-cooled in liquid nitrogen. Thus, crystals can be placed in storage at any point and until the chosen time for diffraction data collection. This gives the crystallographer enormous freedom as to when to set up crystallizations, when in the growing cycle to place crystals in storage and when to use the crystals for diffraction measurement. Clearly, such attractive features of the IMISXcryo approach are in stark contrast to what is offered by methods that require data acquisition at RT. These include injector-based SX at synchrotron and XFEL sources and the original IMISX method. In all cases, beamtime must be available, essentially on-demand, for crystals as they reach maturity, which is not realistic. Alternatively, crystal growth must be controlled to coincide with scheduled beamtime. This will be possible for some proteins, but it will not work with all. Setting up a number of individual crystallizations separated in time guarantees that some would be available for use at the scheduled beamtime, but this is certainly a wasteful process. For injector-based data collection, the large quantities of protein (and ligand when present) needed, in addition to the demands on equipment, could prove to be limiting.

As noted, it is not uncommon for crystals to grow optimally at temperatures other than ambient and for data collection at other than the growth temperature to be problematic. The IMISXcryo method is of real benefit in such cases because wells with crystals growing at non-ambient temperature can be snap-cooled and stored in liquid nitrogen for data collection at 100 K at the convenience of the crystallographer.

#### 4.1.3. Wells work well at 100 K   

The challenge in the current study was to implement a method for retrieving wells containing mesophase and crystals from IMISX plates and to rapidly quench them in liquid nitrogen in preparation for data collection. The COC inner plates developed for IMISX worked nicely in this application. They can be removed individually from the double-sandwich plate used for crystal growth and pin-mounted, excess precipitant can be wicked away from around the mesophase and they can then be snap-cooled in liquid nitrogen for storage and shipping in cryo-vials in Dewars. At the synchrotron beamline, the wells can be mounted on the goniometer by robot for data collection at 100 K. The only speciality item needed to implement the IMISXcryo method is the 2 × 2 mm composite support on the pin of the goniometer base to which the well is attached. As outlined in §[Sec sec2.2.2]2.2.2 (Step 2) and Supplementary Fig. S1, these can be fabricated from readily available materials in minutes.

The original configuration for IMISXcryo had the plastic well glued directly to the pin of the goniometer base. This worked reasonably well but required very careful handling, and occasionally the bond between the pin and the well failed. Another problem arose with this arrangement in that the well could bend ever so slightly in the flow stream of the cryo-jet, causing the crystal to move fractionally in the beam. The square composite support solved both of these problems. The slight curvature in the support extends into the well, providing rigidity and eliminating problems with well bending and crystal movement. The double-stick tape that is part of the composite support provides a very tight bond such that bond failure rarely happens. Further, it makes attachment of the well to the pin extremely simple and fast.

#### 4.1.4. High crystal density for high throughput and low cost   

The traditional method for MX data collection at cryotemperatures uses loops with a single crystal per loop. Thus, a Dewar with five SPINE pucks will typically accommodate 50 crystals. Using the IMISXcryo method, individual wells contain at least 20 and as many as several hundred crystals usually distributed throughout the mesophase bolus. A single Dewar therefore has a minimum capacity of 1000 crystals. This increases throughput enormously because the number and extent of crystal manipulations required for the collection of a complete data set are reduced dramatically. With the large increase in crystal-carrying capacity per Dewar, the numbers of Dewars required per project, as well as shipping costs, which can be considerable, are also reduced.

#### 4.1.5. Flat-well advantage   

The flat, low-profile form of the IMISX wells means that diffraction data can be collected over a very wide angular range (±45° in this study and ±70° in others; unpublished work), making complete data collection from single crystals possible in many cases. This holds true provided that there is no severe preferential crystal orientation (see §[Sec sec4.1.8]4.1.8).

#### 4.1.6. Efficiency of the IMISXcryo method: soluble proteins   

In the original IMISX study performed at RT, lysozyme native and bromine SAD phasing data were assembled from 113 and 239 crystals, respectively. Using the IMISXcryo method, complete native data were obtained from just two crystals, while eight crystals were sufficient for bromine SAD phasing. These values correspond to a 50-fold to 30-fold reduction in the number of crystals required. With porcine insulin, sulfur SAD phasing was achieved by merging 60° of data from each of six crystals in a single well by the IMISXcryo method. Although not a direct comparison, in the original IMISX study 992 crystals were needed for successful sulfur SAD phasing with lysozyme. This dramatic reduction in crystal and protein overheads highlights the impressive efficiencies of the IMISXcryo approach.

#### 4.1.7. Efficiency of the IMISXcryo method: membrane proteins   

Similar advantages in terms of the number of crystals required and the time spent collecting data are apparent for the membrane proteins included in this study. Specifically, with AlgE it was possible to collect a composite 100° data set in 20 min from measurements performed at four locations on a single crystal by the IMISXcryo method. At RT, 175 crystals and 2 h of beamtime were needed. In contrast to AlgE, PepT_St_ is more radiation-sensitive. At RT, only 0.6° of data could be collected per crystal with an attenuated beam to minimize the devastating radiation damage that occurs at ambient temperatures. This resulted in the need to measure 572 crystals in 20 wells to generate a native data set to 2.8 Å resolution. The entire process required more than 10 h of beamtime. Using the IMISXcryo method, the same task was accomplished to a much higher resolution of 2.4 Å using just two crystals and less than 20 min of beamtime. This is comparable to conventional data-collection rates with loop-harvested crystals. Although crystals intrinsically have lower mosaicity at RT [for example, PepT_St_ has mosaicity values of 0.05° and 0.17° at RT (Huang *et al.*, 2015 ▸) and 100 K (this study; Table 2 ▸), respectively], which can enhance the diffraction signal at high scattering angles, the best diffraction resolution achievable at ambient temperature is often limited by radiation damage. Alas, damage is much more severe at these temperatures. For more challenging cases, such as the weakly diffracting microcrystals of GPCRs, it is difficult if not impossible to collect high-resolution data at RT using a synchrotron source. The human β_2_AR crystal structure, which was among the first GPCR structures to be solved, was originally reported at a resolution of 2.4 Å using thirty one 10–20° data wedges merged from 27 crystals (Cherezov *et al.*, 2007 ▸). This same target was used in the current study to further test the IMISXcryo method. IMISX wells that contained β_2_AR crystals typically had tens of to a few hundred needle-shaped crystals in each well with maximum diameters of 5–10 µm. On average, 20–50 crystals from good wells yielded useful diffraction data. In one instance, 149 partial data sets were collected from a single well, of which 104 could be processed, resulting in a complete merged data set to 2.5 Å resolution. Compared with the originally published 2.4 Å resolution structure, this slightly poorer resolution and the threefold larger number of crystals required may reflect the smaller crystal sizes available for measurement in the current study. The quality of the protein preparation, which is variable and difficult to control, also impacts on crystal quality and may well have been a contributing factor.

#### 4.1.8. Preferential crystal orientation, multiplicity and completeness   

**Figure 5:**
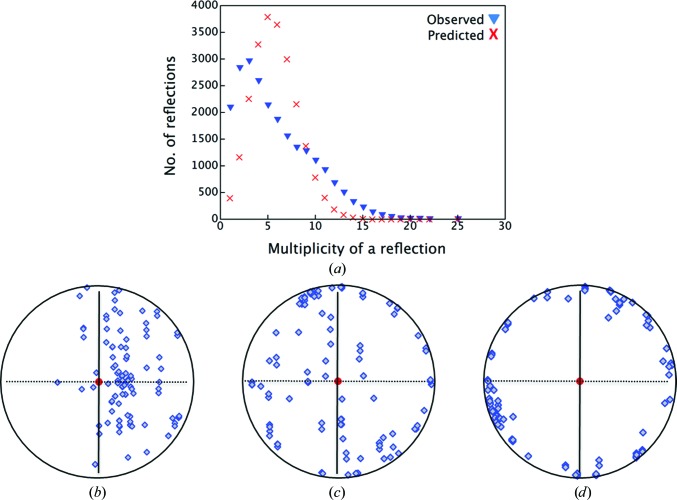
Distribution of reflection multiplicity and crystal orientation in an IMISXcryo data set recorded using 104 β_2_AR crystals. (*a*) Blue, observed multiplicity. Red, binomial distribution of multiplicity predicted for an effective crystal rotation range of 2.5°. (*b*) Projection of the unit-cell *a* axis along the X-ray beam direction (red dot). The horizontal axis (dotted line) is the rotation-spindle direction and the vertical axis (solid line) is perpendicular to both the X-ray beam and rotation-spindle directions. Each blue diamond represents one crystal. For any given crystal in the set, the angle between its unit-cell axis and the X-ray beam direction is indicated by the position of the corresponding diamond on a circle. These range from 90° on the limiting circle to 0 or 180° at the centre of the circle. (*c*) Projection of the unit-cell *b* axis along the X-ray beam direction. (*d*) Projection of the unit-cell *c* axis along the X-ray beam direction. The skewed distribution in (*a*) is consistent with the preferential crystal orientation with the *a* axis of the unit cell close to the X-ray beam direction (*b*).

The multiplicity of reflections in a merged set of many partial data sets from randomly oriented crystals follows a binomial distribution (Huang *et al.*, 2015 ▸). The merged data for β_2_AR crystals recorded in the current study consisted of 104 partial data sets. In this case, however, the multiplicity displayed a departure from the predicted binomial distribution, with a peak at lower multiplicity values and a shoulder at higher multiplicity values than the binomial mean (Fig. 5 ▸ *a*). This anomalous behaviour is attributed to preferential crystal orientation, which can lead to incomplete data sets where insufficient data are collected for certain reflections. Typical β_2_AR crystals were needle-shaped, with a length of 20 µm and a diameter of 5–10 µm. With considerable frequency, the crystals were found growing with the long axis of the crystal, which corresponds to the crystallographic *c* axis, in the plane of the IMISX well (Figs. 5 ▸ *b*, 5 ▸ *c* and 5 ▸ *d*). As expected, therefore, analysis of crystal orientation with respect to the beamline coordinates showed that the direction of the unit-cell *c* axis was heavily weighted into the plane perpendicular to the X-ray beam direction (Fig. 5 ▸ *d*). This preferential orientation does not compromise data-set completeness provided that the two other crystal axes are randomly orientated. With β_2_AR crystals, however, the *a* axis tended to lie normal to the IMISX plate and along the X-ray beam direction in the diffraction geometry used for most data collections in this study. These observations are consistent with the anomalous multiplicity distribution in Fig. 5 ▸.

In light of the above, there are several measures that might be implemented to deal with preferential orientation as observed in the current application: (i) collect data with the IMISX plate at various inclinations to the X-ray beam to increase angular coverage, (ii) perform grid scans to identify (and to use in data collection) crystals with an edge oriented towards the X-ray beam, (iii) monitor orientation coverage during serial data collection (Leslie & Powell, 2007 ▸; Pothineni *et al.*, 2014 ▸; Zeldin *et al.*, 2015 ▸) and improve it if possible, (iv) increase the total number of crystals/data sets to compensate for the slight net reduction in data quality and (v) use a thicker well spacer to provide a less confining and preferentially orienting mesophase environment in which to grow crystals. Unfortunately, this last measure comes at the cost of reduced signal to noise in the data owing to X-ray scatter and absorption by the additional mesophase.

In circumstances where there is an abundance of crystals and beamtime, the fourth measure on the list above is the simplest and may well suffice. When crystals and/or beamtime are limiting, however, the objective should be to collect data of the highest possible completeness as opposed to gathering more partial data sets that contribute to an anomalous multiplicity profile. This goal can best be realised by implementing one or all of the first three measures listed above. Parenthetically, we note that in the early stages of the β_2_AR project described here only one in about ten wells contained crystals with reasonable diffraction. These provided merged data sets with less completeness. Searching for good wells was time-consuming and the possibility loomed that the sample would run out. In hindsight, better data might have been collected with the IMISX plate in various inclinations towards the beam direction for increased angular coverage and a speedier realisation of high completeness. With better monitoring and intervention during data collection, complete data of high multiplicity and quality should be attainable with fewer crystals.

#### 4.1.9. Comparison with injector-based serial crystallo­graphy   

Since the advent of the XFEL, SX has emerged as an alternative method for high-resolution structure determination from microcrystals. To date, the lipid cubic and sponge phases have proven to be effective as delivery media for both *in situ*-grown crystals and for extant crystals grown in other media in injector-based SX applications. Injector-based delivery was originally developed for SX with an XFEL (Johansson *et al.*, 2012 ▸; Liu *et al.*, 2013 ▸; Caffrey *et al.*, 2014 ▸; Weierstall *et al.*, 2014 ▸; Fenalti *et al.*, 2015 ▸; Kang *et al.*, 2015 ▸). It was subsequently adapted for use at synchrotron sources (Botha *et al.*, 2015 ▸; Nogly *et al.*, 2015 ▸). Although not strictly an *in situ* method as currently implemented, the injector-based SX approach has the advantage of continuous crystal delivery. However, control over which data are collected when and where is limited, resulting in low hit rates and wasted sample. Further, the method yields still images, which present challenges at the data-processing stage. The injectors currently in use only work at RT. As with the original IMISX method, which also uses RT data collection, problems will arise with such ambient data-collection methods when the target protein only crystallizes at temperatures other than RT. With an XFEL source, data collection at RT is generally not a problem because diffraction information can be gathered ahead of significant X-ray damage. This is not the case with synchrotron X-rays, where radiation damage at RT is severe. In distinct contrast, the IMISXcryo method introduced here offers full control over data collection, which can be tailored to crystals of different sizes, shapes and radiation-sensitivity and, in the crystal-picking mode, has a perfect hit rate. The method generates conventional rotation data with the possibility of rastering to locate diffracting crystals with minimal radiation damage. Further, the large number of crystals per sample and the fact that snap-cooled wells can be stored in Dewars and used with wet-mounting automatic sample changers (with vials) at synchrotron beamlines makes IMISXcryo a generally useful and accessible high-throughput method that is extremely efficient in terms of amount of protein, lipid and ligand required. An added feature is that the IMISX plates can be used for fixed-target SX with an XFEL source at ambient or cryogenic temperatures.

#### 4.1.10. Crystal visibility   

Loop-harvested crystals, snap-cooled in liquid nitrogen, usually require diffraction-based rastering to find the crystals, which lie hidden from view in an opaque mesophase. The rastering and image-inspection processes take time and rastering can damage the crystals. In this regard, then, the IMISXcryo method offers a considerable advantage. We find that crystals are generally visible in the mesophase bolus in snap-cooled IMISXcryo wells when viewed with the in-line microscope (Fig. 2 ▸). This is a real plus because it provides rapid and efficient crystal picking, ensuring a 100% hit rate.

#### 4.1.11. Nanograms to micrograms of protein are required   

As noted, the IMISXcryo method is extremely efficient in terms of numbers of crystals, and thus the amount of protein (and ligand when present), required to solve a structure. Some numbers are worth considering (Table 4 ▸). MR phasing was possible with a single crystal of AlgE and with two crystals of PepT_St_ by the IMISXcryo method. 104 crystals were needed in the case of β_2_AR. For all three proteins, the crystals used were contained within a single mesophase bolus in a single well. With DgkA, which typically grows crystals at low density, 12 crystals in five wells were required to complete the data set. Regardless of protein target, the amount of protein required for these assorted measurements ranged from as little as 250 ng in the case of AlgE and PepT_St_ to 1.5 µg in the case of DgkA. These amounts are orders of magnitude less than those used for structure determination of *in meso*-grown membrane protein crystals by injector-based SX with synchrotron and FEL X-rays. In addition to its efficiency, what makes the IMISXcryo method so attractive is the fact that all data collection is performed directly in the well in which the crystals grow without the need to harvest them from the sticky, viscous mesophase. It is only necessary to open the double-sandwich plate, to remove the relevant inner well and to mount and snap-cool it in liquid nitrogen, whereupon it is ready for data collection. The entire process can be completed in minutes.

#### 4.1.12. Radiation damage   

One of the major advantages of the IMISXcryo method relates to the fact that the diffraction measurements are made at 100 K. This means that compared with data collection at RT, crystal lifetimes are extended by factors of 20-fold to 50-fold. Limiting accumulated doses, after which severe damage sets in, range from 0.2 to 0.5 MGy at RT and from 10 to 20 MGy at 100 K (Holton, 2009 ▸). With less damage, the amount of data that can be collected from a single crystal increases dramatically. Consequently, the number of crystals required for a complete data set is reduced significantly. Individual data sets with sufficient angular coverage and a minimum of radiation damage provide better data integration and a final merged data set of high quality, which is important for experimental phasing. Estimated accumulated doses for all crystals included in this study are summarized in Table 1 ▸.

#### 4.1.13. Data-set selection for scaling and merging   

As explained in §[Sec sec2]2, throughout this study the ‘best’ data sets were selected for scaling and merging. This ‘cherry-picking’ approach was possible because in contrast to our previous IMISX RT studies (Huang *et al.*, 2015 ▸) every IMISXcryo data set (Table 2 ▸) covered either a substantial part of reciprocal space or at least a few degrees owing to an approximately 50-fold tolerance of X-ray dose before radiation damage set in. This resulted in merged data sets with completeness and multiplicity values comparable to those of single data sets collected using conventional methods. When applied, the correlation coefficient-based selection of data sets for good agreement was successful in retaining data sets that were sufficiently isomorphous to each other. Further optimization of data-set selection and detailed analysis of the scaling and merging results are in progress.

### 4.2. Future prospects   

In the following, some ideas for the further development of the IMISXcryo method are discussed.

#### 4.2.1. X-ray facility support   

What we report here amounts to a demonstration study of the IMISXcryo method performed with four integral membrane proteins and two soluble proteins. Because of its success, the method is now being used in projects in the Membrane Structural and Functional Biology group on a regular basis. The method has recently been demonstrated at a workshop held at the Swiss Light Source (http://indico.psi.ch/conferenceDisplay.py?confId=3677). We are now working to refine and improve all aspects of the IMISXcryo method to make it simple, robust, reproducible, convenient and inexpensive to use. The adoption of the method by the crystallo­graphic community will certainly benefit from support for *in situ* work at synchrotron facilities through the provision of appropriate beamline plate hotels/imagers/incubators, sample-handling robots, goniometers, in-line microscopes and software.

#### 4.2.2. Commercial availability   

In the interest of convenience and reproducibility, the materials used to make IMISXcryo measurements, as described in this work, should be available commercially. Two options are likely to emerge. Firstly, the component parts will be purchased individually, cut to size and assembled manually. This is the approach that was used in the current study. Secondly, the various components, cut to size and pre-assembled, will be available commercially. MiTeGen has an IMISX plate available for purchase. Gonio­meter bases with pins and supports attached for quick, easy and durable mounting of wells prior to snap-cooling, or variants of the same, are likely to soon find suppliers.

#### 4.2.3. Optimizing well handling and snap-cooling   

Since the IMISXcryo method does not involve a potentially damaging crystal-harvesting step, the expectation was that such crystals should provide as good or better diffraction data than loop-harvested crystals. Generally, we found this to be the case. However, obtaining substantially better IMISXcryo diffraction data was not our experience. It is possible, therefore, that the processes of harvesting wells from IMISX plates, removing precipitant from around the bolus to lower the thermal mass for faster cooling and snap-cooling in liquid nitrogen will need optimizing. What we seek is ultrafast and reproducible bolus cooling to liquid-nitrogen temperatures to generate an optically clear, vitrified mesophase so as to preserve uncompromised the native diffraction quality of the crystals in their natural growth environment. An optically clear mesophase is a real plus because crystals can be easily and quickly hand-picked on-line or off-line for most efficient diffraction data collection. Instruments are available for the automated ‘plunge-freezing’ of thin-layer samples on grids into liquid cryogens, such as ethane at liquid-nitrogen temperatures, for cryo-electron microscopy (Tivol *et al.*, 2008 ▸). These will be investigated to determine whether more uniform IMISXcryo samples of improved quality can be prepared conveniently and are worth investing in.

The sponge phase is a more fluid and hydrated variant of the cubic phase (Cherezov *et al.*, 2006 ▸). Many protein targets produce crystals in the sponge phase (Caffrey, 2015 ▸). The sponge phase is characterized by having a considerably lower viscosity than that of the cubic phase from which it derives. Handling the sponge phase in IMISX plates requires particular care, especially during precipitant removal from the well immediately prior to snap-cooling. Making this process less prone to failure and more user-friendly is an objective that is worth pursuing.

#### 4.2.4. Crystal picking off-line   

Valuable beam time can be saved by performing the crystal-selection or crystal-picking step off-line. Fiducials will be needed on individual wells with which to align the sample in the beam for subsequent serial diffraction data collection. With automated data gathering, the method lends itself to an entirely automated process with little or no human intervention for the most efficient use of valuable beam time and other resources. Automation could usefully be extended to the evaluation of diffraction data in, or in close to, real time to ensure that sufficient, but not too much, data of the right quality have been collected for the task at hand. Ideally, such measurements should be possible *via* remote access (Pothineni *et al.*, 2014 ▸).

#### 4.2.5. Fast crystal selection   

An alternative to hand picking crystals is to use fast, fixed or helical line-scanning as implemented for SX measurements at synchrotron (Gati *et al.*, 2014 ▸) and FEL (Hunter *et al.*, 2014 ▸) sources. While this might speed up diffraction data collection and reduce beam-time requirements, the burden shifts to needing efficient data acquisition, storage, processing and analysis, the latter two ideally in live time.

For some proteins we have found that all crystals in a given well were either of good or of poor quality. With β_2_AR in the current study, for some trials only one in 10–20 wells had good crystals. A more efficient method, such as grid-scanning, to identify quality wells is required. With β_2_AR, we could not rely on the visual appearance of the crystals; they all looked uniformly good. Generally, however, larger crystals gave more and better data.

#### 4.2.6. Thinner samples and windows, and alternative window materials   

With weakly scattering crystals and for demanding crystallographic measurements, such as native SAD phasing, the signal to noise must be optimized. For IMISXcryo, as implemented here, this can be realised by using thinner samples and window materials for lower beam attenuation and scatter. With crystals that typically range from 10 to 30 µm in maximum dimension, the mesophase thickness could likely be reduced from 140 µm, as used in this study, by a factor of two without compromise. However, any reductions to where the mesophase thickness approaches the crystal size would have to be performed with due recognition that it could lead to preferential crystal orientation, with negative consequences for data completeness (§[Sec sec4.1.8]4.1.8). Thinning the COC sheet further from its current thickness of 25 µm is an option, but it will mean that the windows are that much more porous. As alternatives, graphene and silicon nitride windows, both of which are intrinsically watertight and can be made extremely thin, are being investigated.

#### 4.2.7. A glass alternative   

The IMISXcryo plate can be viewed as a plate within a plate. The outer plate is a glass sandwich similar in construction to the original *in meso* sandwich plates. The reasons for using glass in both applications reflect its many advantages. Glass is watertight, optically clear, nonbirefringent, readily available and inexpensive. Depending on the glass, it can be more or less UV-transmitting and thus more or less useful for UV fluorescence imaging. Disadvantages include the need for a glass-cutting tool with which to open plates and careful handling to prevent injury. Opening individual wells in standard glass sandwich plates requires practice, patience and skill. However, when working with IMISX plates, cutting the outer glass plate to expose the inner plastic sandwich is quite straightforward. Still, careful handling and eye protection are required. For these reasons, having an alternative outer sandwich material is desirable. Several are being investigated. Regardless of the material, it must provide a distinct advantage on balance over glass to be a serious contender.

#### 4.2.8. Laboratory-based X-ray sources   

While the focus in this study has been on synchrotron sources, it is expected that the IMISXcryo method will prove useful with laboratory-based X-ray diffractometers. The latest models come with impressive brightness, flux and reduced beam sizes. At a minimum, therefore, *in situ* screening for crystal hits should be possible, with showers of microcrystals giving rise to powder patterns. In the best of circumstances, structures may well be accessible. The real benefit, of course, comes from being able to make the measurements at cryogenic temperatures at home and to reuse the pre-screened wells at a synchrotron source for higher resolution data collection.

#### 4.2.9. XFEL sources   

The IMISX plates should also prove to be useful in fixed-target applications at XFEL sources (Hunter *et al.*, 2014 ▸). Serial femtosecond crystallographic (SFX) measurements can be made in scanning mode with sufficient spatial separation between shots to avoid radiation-damage spillover. Alternatively, the location of the crystals in the bolus can be identified off-line, enabling directed data collection with the XFEL and a 100% hit rate. While measurements with an XFEL beam are considered to incur little substantive radiation damage, the option is there to use the IMISX samples at ambient and at cryogenic temperatures.

#### 4.2.10. Soluble proteins   

The *in meso* crystallization method works with soluble proteins. In the current study lysozyme and insulin were used as model soluble proteins, and for the purposes of evaluating the IMISXcryo method crystals were grown *in meso* in IMISX plates. It is also possible to use the cubic mesophase as an inert, viscous medium in which to suspend extant crystals of soluble proteins for delivery or presentation to an interrogating X-ray beam. This approach, and variations on it (Sugahara *et al.*, 2015 ▸), have been used to advantage for SX at synchrotron and XFEL sources. The same method can be implemented for IMISXcryo, where the attractive features of SX data collection, which works with micrometre-sized crystals, are exploited.

#### 4.2.11. Ligand-binding screening   

Currently, ligand-binding screening assays performed *in meso* require the merging of data collected from as many as 50–100 crystals to obtain a structure with recognizable ligand density. Each one of these crystals must be harvested individually from plates, snap-cooled and diffraction-rastered to locate and to centre the crystal for data collection. Given the inefficiency of the harvesting process, this translates to several hundreds of wells that must be opened and from which crystals must be harvested tediously. The process may need to be repeated at different ligand concentrations, with the result that many hundreds of wells with crystals may need to be processed to obtain a useful result. This, in turn, could translate to having to set up certainly tens, and possibly hundreds, of plates. With the IMISXcryo method, it should be possible to collect the requisite data directly from the crystal manifest of just one to several wells without the need to harvest. Thus, a single 96-well plate could provide highly efficient, crystal structure-based screening of 10–20 ligands.

#### 4.2.12. Towards even smaller crystals   

The bulk of the membrane-protein crystals used in this study ranged from 20 to 30 µm in the maximum dimension. While these are small, being able to work with even smaller crystals, of the type that often emerge as initial hits, would be advantageous. For such an application, thinner boluses and thinner windows for improved signal to noise would be desirable. The provision of a smaller, higher flux beam would also be important. These advances, coupled with faster detectors, would support measurements of unprecedented quality and enable measurements in the crystal size domain of 2 µm, where photoelectron escape is possible (Sanishvili *et al.*, 2011 ▸), and in the time interval before the consequences of radiation damage become detrimental. Such developments are in progress.

In summary, the IMISXcryo method provides in a simple, double-sandwich plate a means for high-throughput membrane-protein and soluble-protein crystallization over a range of temperatures, not just ambient. The snap-cooled wells containing the crystal-laden mesophase can be placed in liquid nitrogen for long-term storage without the need for added cryoprotectants. The method is compatible with synchrotron and XFEL-based collection at cryogenic temperatures of diffraction data of a quality suitable for SAD phasing. The IMISXcryo approach is extremely efficient in that nanogram to single-digit microgram quantities of protein suffice for high-throughput measurements made directly *in situ* without the need to harvest crystals from the viscous and sticky hosting mesophase.

## Supplementary Material

PDB reference: insulin-N, IMISX RT, 5d52

PDB reference: IMISXcryo, 5d53

PDB reference: loop, 5d54

PDB reference: DgkA, IMISXcryo, 5d56

PDB reference: loop, 5d57

PDB reference: PepT_st_, IMISXcryo, 5d58

PDB reference: loop, 5d59

PDB reference: β_2_AR, IMISXcryo, 5d5a

PDB reference: loop, 5d5b

PDB reference: lyso-N, IMISXcryo, 5d5c

PDB reference: AlgE, IMISXcryo, 5d5d

PDB reference: insulin-S, IMISXcryo, 5d5e

PDB reference: lyso-Br, IMISXcryo, 5d5f

Supporting Information.. DOI: 10.1107/S2059798315021683/wa5105sup1.pdf

Supplementary Movie S1. The materials and the method used to set up and to use IMISX plates.. DOI: 10.1107/S2059798315021683/wa5105sup3.mp4

Supplementary Movie S2. The materials and the method used to assemble the composite support for the IMISX well.. DOI: 10.1107/S2059798315021683/wa5105sup4.mp4

Supplementary Movie S3. The materials and the methods used to remove the well from an IMISX plate, to mount it on a goniometer base and to snap-cool the sample in liquid nitrogen.. DOI: 10.1107/S2059798315021683/wa5105sup5.mp4

Supplementary Movie S4. The sample-handling robot on beamline PX I at the SLS moving IMISXcryo samples between the Dewar and the goniometer.. DOI: 10.1107/S2059798315021683/wa5105sup6.mp4

## Figures and Tables

**Table 1:** Facts and figures used to estimate the accumulated dose per crystal for the IMISXcryo and IMISX RT studies

Experiment	IMISXcryo								IMISX RT
Protein	Insulin-N	Lyso-N	AlgE	PepT_St_	β_2_AR	DgkA	Insulin-S	Lyso-Br	Insulin-N
Crystal size (µm)	30 × 30 × 30	15 × 15 × 30	15 × 15 × 150	20 × 20 × 30	5 × 10 × 30	20 × 20 × 50	30 × 30 × 30	15 × 15 × 30	30 × 30 × 30
Wavelength (Å)	0.97857	1	0.97856	0.97852	1.0332	1	2.06643	0.91881	1
Flux (photons s^−1^)	5.9 × 10^10^	8.0 × 10^10^	5.0 × 10^11^	7.4 × 10^10^	3.2 × 10^11^	1.8 × 10^11^	4.3 × 10^10^	3.4 × 10^10^	2.0 × 10^11^
Beam size (µm)	10 × 18	10 × 30	15 × 20	10 × 18	10 × 18	10 × 18	15 × 20	10 × 30	10 × 30
Estimated dose rate† (MGy s^−1^)	0.157	0.133	0.798	0.197	0.937	0.492	0.306	0.096	0.333
Oscillation per exposure (deg s^−1^)	0.4/0.2	0.1/0.1	0.1/0.1	0.1/0.3	0.1/0.3	0.1/0.1	0.1/0.1	0.1/0.1	0.2/0.05
Oscillation range per crystal (°)	60	40	20, 25, 25, 30	60	3	20–40	60	30	5
Exposure time per crystal (s)	30	40	20, 25, 25, 30	180	9	20–40	60	30	1.25
Estimated accumulated dose per crystal† (MGy)	4.7	5.3	16.0–23.9	35.4	8.4	9.8–19.7	18.4	2.9	0.42
Total No. of crystals	1	2	1	2	104	12	6	8	25
Total angular range per data set (°)	60	80	100	120	312	280	360	240	125
Total exposure time per data set (s)	30	80	100	360	936	280	360	240	31.25
Total dose per data set (MGy)	4.7	10.6	79.8	70.8	877	137.8	110.2	23	10.4

† Estimate based on equation (5) in Holton (2009 ▸). For lyso-Br crystals at the Br *K* edge, the ‘dose-doubling’ effect of bromide has been accounted for.

**Table 2:** Data-collection and refinement statistics for IMISX samples All data-processing statistics are reported with Friedel pairs merged except for insulin-S and lyso-Br. Values in parentheses are for the highest resolution shell.

	Insulin-N	Insulin-N	Lyso-N	AlgE	PepT_St_	β_2_AR	DgkA	Insulin-S	Lyso-Br
PDB code	5d52	5d53	5d5c	5d5d	5d58	5d5a	5d56	5d5e	5d5f
Crystal-growth temperature (K)	293	293	293	293	293	293	277	293	293
Data collection									
Temperature (K)	293	100	100	100	100	100	100	100	100
Phasing method	MR	MR	MR	MR	MR	MR	MR	S-SAD	Br-SAD
Space group	*I*2_1_3	*I*2_1_3	*P*4_3_2_1_2	*P*2_1_2_1_2_1_	*C*222_1_	*C*2	*P*2_1_2_1_2_1_	*I*2_1_3	*P*4_3_2_1_2
Unit-cell parameters									
*a*, *b*, *c* (Å)	79.71, 79.71, 79.71	78.36, 78.36, 78.36	77.70, 77.70, 38.94	46.33, 66.35, 176.84	100.16, 109.52, 111.47	108.04, 170.58, 40.44	75.61, 93.19, 142.74	78.20, 78.20, 78.20	78.28, 78.28, 38.12
α, β, γ (°)	90, 90, 90	90, 90, 90	90, 90, 90	90, 90, 90	90, 90, 90	90, 106.30, 90	90, 90, 90	90, 90, 90	90, 90, 90
Unit-cell volume (Å^3^)	506452	481153	235092	543605	1222773	745287	1005760	478212	233590
Wavelength (Å)	1.0	0.97857	1	0.97856	0.97852	1.0332	1	2.06643	0.91881
ISa threshold	5	—	—	—	—	5	3	3	—
CC_set_ cutoff	—	—	—	—	—	—	0.9	0.9	—
No. of crystals	25	1	2	1	2	104	12	6	8
Resolution (Å)	50–1.80 (1.85–1.80)	50–1.50 (1.54–1.50)	50–1.70 (1.74–1.70)	50–2.40 (2.46–2.40)	50–2.40 (2.46–2.40)	50–2.50 (2.57–2.50)	50–2.80 (2.87–2.80)	50–2.40 (2.46–2.40)	50–1.50 (1.54–1.50)
*R*_meas_	0.295 (2.354)	0.123 (1.420)	0.195 (1.650)	0.162 (1.186)	0.122 (0.999)	0.203 (2.084)	0.244 (2.982)	0.118 (0.712)	0.250 (2.233)
*R*_p.i.m._ †	0.081 (0.652)	0.049 (0.579)	0.082 (0.692)	0.086 (0.612)	0.059 (0.487)	0.085 (0.879)	0.080 (1.012)	0.030 (0.309)	0.746 (0.688)
〈*I*/σ(*I*)〉	6.96 (0.88)	9.08 (1.21)	6.16 (1.10)	5.98 (1.45)	9.31 (2.18)	7.27 (1.11)	4.42 (1.00)	19.50 (2.00)	6.39 (0.97)
Completeness (%)	100 (100)	99.9 (100)	99.7 (100)	92.6 (90.8)	99.4 (98.8)	95.1 (91.0)	98.8 (98.6)	99.9 (99.5)	99.4 (92.0)
Multiplicity	13.2 (13.0)	6.4 (6.0)	5.5 (5.7)	3.6 (3.8)	4.3 (4.2)	5.7 (5.6)	9.3 (8.7)	16.0 (5.3)	11.2 (10.5)
CC_1/2_	0.99 (0.46)	0.99 (0.46)	0.99 (0.32)	0.99 (0.62)	0.99 (0.62)	0.99 (0.21)	0.99 (0.36)	0.998 (0.86)	0.98 (0.38)
CC_anom_ ‡	—	—	—	—	—	—	—	0.47	0.44
Mosaicity§ (°)	0.11	0.14	0.09	0.12	0.17	0.12	0.25	0.16	0.12
Refinement									
Resolution (Å)	39.85–1.80	38.76–1.50	39.55–1.70	25.22–2.40	45.68–2.40	43.96–2.50	46.52–2.80	27.70–2.41	35.06–1.50
No. of reflections	7975	13002	13594	20520	24159	23086	25188	6025	35932
*R*_work_/*R*_free_	0.17/0.21	0.16/0.17	0.18/0.22	0.22/0.26	0.21/0.25	0.21/0.26	0.24/0.28	0.17/0.22	0.18/0.21
No. of atoms									
Protein	414	814	1019	3222	3475	3546	4444	414	1019
Ligand/ion	10	37	21	233	502	210	260	21	19
Water	30	63	96	23	64	53	13	31	141
*B* factors (Å^2^)									
Protein	24.59	21.10	22.36	49.16	55.52	64.45	93.72	33.04	20.11
Ligand/ion	41.86	47.34	35.28	68.58	71.79	86.14	113.75	53.68	30.53
Water	36.05	31.63	27.14	46.03	53.43	61.90	89.98	41.49	27.79
R.m.s. deviations									
Bond lengths (Å)	0.012	0.009	0.008	0.010	0.005	0.006	0.004	0.002	0.007
Bond angles (°)	1.094	1.045	1.022	1.220	0.932	1.487	0.580	0.514	1.038
Ramachandran plot									
Favoured (%)	98.0	98.0	99.24	95.70	98.19	98.62	99.65	98.00	98.47
Allowed (%)	2.0	2.00	0.76	4.00	1.81	1.38	0.35	2.00	1.53
Outliers (%)	0	0	0	0.30	0	0	0	0	0
*MolProbity* clashscore	3.6	3.5	5.4	6.6	9.1	5.5	6.8	2.4	2.5

† *R*_p.i.m._ was calculated as *R*_meas_/(multiplicity)^1/2^.

‡ Anomalous correlation coefficient evaluated with data truncated to 2.4 Å resolution for insulin-S and to 2.0 Å resolution for lyso-Br.

§ For IMISX data, mosaicity is reported as the median over all crystals.

**Table 3:** Data-collection and refinement statistics for reference loop-harvested crystals at 100 K All data-processing statistics are reported with Friedel pairs merged. Values in parentheses are for the highest resolution shell.

	Insulin-N	PepT_St_	β_2_AR	DgkA
PDB code	5d54	5d59	5d5b	5d57
Crystal-growth temperature (K)	293	293	293	277
Data collection				
Phasing method	MR	MR	MR	MR
Space group	*I*2_1_3	*C*222_1_	*C*2	*P*2_1_2_1_2_1_
Unit-cell parameters				
*a*, *b*, *c* (Å)	77.51, 77.51, 77.51	101.57, 110.25, 110.34	106.73, 170.42, 40.30	75.32, 91.34, 143.36
α, β, γ (°)	90, 90, 90	90, 90, 90	90, 105.94, 90	90, 90, 90
Unit-cell volume (Å^3^)	465665	1235598	733014	986278
Wavelength (Å)	1.03321	1.03321	1.03321	1
No. of crystals	1	1	2	1
Resolution (Å)	50–1.50 (1.54–1.50)	50–2.40 (2.46–2.40)	50–3.80 (3.90–3.80)	50–2.80 (2.87–2.80)
*R*_meas_	0.057 (1.127)	0.097 (0.745)	0.526 (1.676)	0.189 (0.953)
*R*_p.i.m._ †	0.022 (0.453)	0.047 (0.357)	0.205 (0.783)	0.076 (0.402)
〈*I*/σ(*I*)〉	15.45 (1.49)	12.00 (2.26)	3.76 (0.96)	7.71 (1.61)
Completeness (%)	99.9 (100)	99.8 (99.9)	99.6 (98.8)	99.6 (98.9)
Multiplicity	6.5 (6.2)	4.4 (4.36)	6.5 (4.6)	6.2 (5.6)
CC_1/2_	0.99 (0.55)	0.99 (0.68)	0.97 (0.23)	0.99 (0.64)
Mosaicity (°)	0.07	0.07	0.20	0.08
Refinement				
Resolution (Å)	38.76–1.50	46.13–2.40	49.62–3.80	45.67–2.80
No. of reflections	12580	24537	6835	24904
*R*_work_/*R*_free_	0.18/0.19	0.21/0.23	0.24/0.27	0.22/0.27
No. of atoms				
Protein	814	3475	3546	4490
Ligand/ion	37	503	186	352
Water	39	75	0	42
*B* factors (Å^2^)				
Protein	34.84	51.78	79.42	57.01
Ligand/ion	59.25	67.15	94.72	79.69
Water	44.43	47.69	0	51.50
R.m.s. deviations				
Bond lengths (Å)	0.006	0.006	0.006	0.004
Bond angles (°)	0.920	1.028	1.144	0.802
Ramachandran plot				
Favoured (%)	98.00	98.64	97.94	98.62
Allowed (%)	2.00	1.36	2.06	1.38
Outliers (%)	0	0	0	0
*MolProbity* clashscore	2.4	7.4	5.6	6.7

† *R*_p.i.m._ was calculated as *R*_meas_/(multiplicity)^1/2^.

**Table 4:** Sample consumption and diffraction measurement statistics

Temperature (K)	100				293	100							
Presentation	Loop				*In situ*	*In situ*							
Protein	Insulin-N	PepT_St_	β_2_AR	DgkA	Insulin-N	Insulin-N	Lyso-N	AlgE	PepT_St_	β_2_AR	DgkA	Insulin-S	Lyso-Br
Lipid	9.9	7.8	9.9 + C†	7.8	9.9	9.9	9.9	7.8	7.8	9.9 + C†	7.8	9.9	9.9
Mesophase per well (nl)	200	50	30	50	200	200	200	50	50	30	50	200	200
Lipid/protein solution (by volume)	3/2	1/1	3/2	1/1	3/2	3/2	3/2	1/1	1/1	3/2	1/1	3/2	3/2
Protein concentration (mg ml^−1^)	35	10	48	12	35	35	50	10	10	48	12	35	50
No. of wells	1	1	1	1	2	1	1	1	1	1	5	1	1
No. of crystals	1	1	2	1	214	1	2	1	2	149	44	14	8
No. of useful crystals	1	1	2	1	25	1	2	1	2	104	12	6	8
Lipid (nl)	120	25	18	25	240	120	120	25	25	18	125	120	120
Protein (µg)	2.8	0.25	0.576	0.3	5.6	2.8	4	0.25	0.25	0.576	1.5	2.8	4
Total angular range per crystal (°)	60	120	120, 240‡	180	5	60	50	40	60, 90‡	3	20, 30, 40‡	60	40
Useful angular range per crystal (°)	60	120	120, 240§	180	5	60	40	20, 25, 30§	60	3	20, 30, 40§	60	30
Indexing rate¶ (%)	100	100	100	100	11.7	100	80	62.5	80	69.8	25.3	42.9	75
Oscillation per frame (°)	0.1	0.1	0.2	0.2	0.2	0.4	0.1	0.1	0.1	0.1	0.1	0.1	0.1
Exposure time per frame (s)	0.1	0.1	0.1	0.1	0.05	0.2	0.1	0.1	0.3	0.3	0.1	0.1	0.1
Total angular range (°)	60	120	360	180	125	60	80	100	120	312	280	360	240

† The lipid shown refers to the hosting MAG. C indicates that cholesterol was included as an additive lipid.

‡ The total angular range measured varied from crystal to crystal.

§ The angular range used in processing varied from crystal to crystal.

¶ The indexing rate was calculated as follows: 100 × [(No. of useful crystals) × (useful angular range per crystal)]/[(No. of crystals) × (total angular range per crystal)].
