# Supplementary material for: In meso in situ serial X-ray crystallography of soluble and membrane proteins at cryogenic temperatures
Source: Acta Crystallogr D Struct Biol. 2016 Jan 1;72(Pt 1):93–112. doi: 10.1107/S2059798315021683 (PMC4756617; doi:10.1107/S2059798315021683)
Supplement: Supplementary file 1 [file d-72-00093-sup1.pdf]

STRUCTURAL
BIOLOGY

Volume 72 (2016)

Supporting information for article:

In meso in situ serial X-ray crystallography of soluble and membrane proteins at cryogenic temperatures

Chia-Ying Huang, Vincent Olieric, Pikyee Ma, Nicole Howe, Lutz Vogeley, Xiangyu Liu, Rangana Warshamanage, Tobias Weinert, Ezequiel Panepucci, Brian Kobilka, Kay Diederichs, Meitian Wang and Martin Caffrey

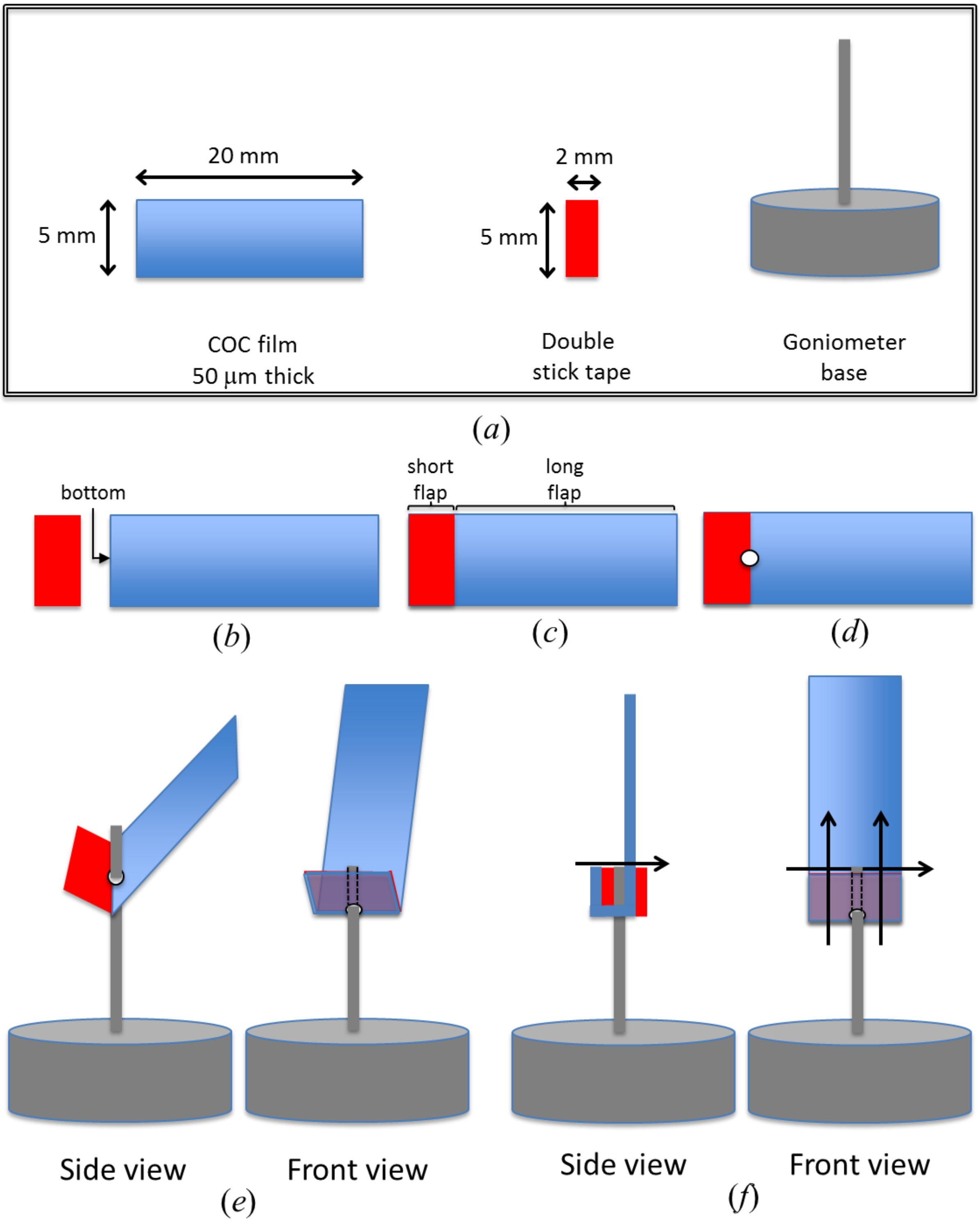

Figure S1 Materials and method for assembling a composite support on a goniometer base for IMISX well mounting. (*a*) Materials used in the assembly. (*b*-*f*) The assembly process as detailed under Methods (Section 2.2.2. Step 2). The parts of the COC film referred to under Methods (Section 2.2.2.) are labelled in (*b*) and (*c*). The arrows in (*f*) indicate where the support is trimmed to a final size of 2 mm × 2 mm. Not drawn to scale.

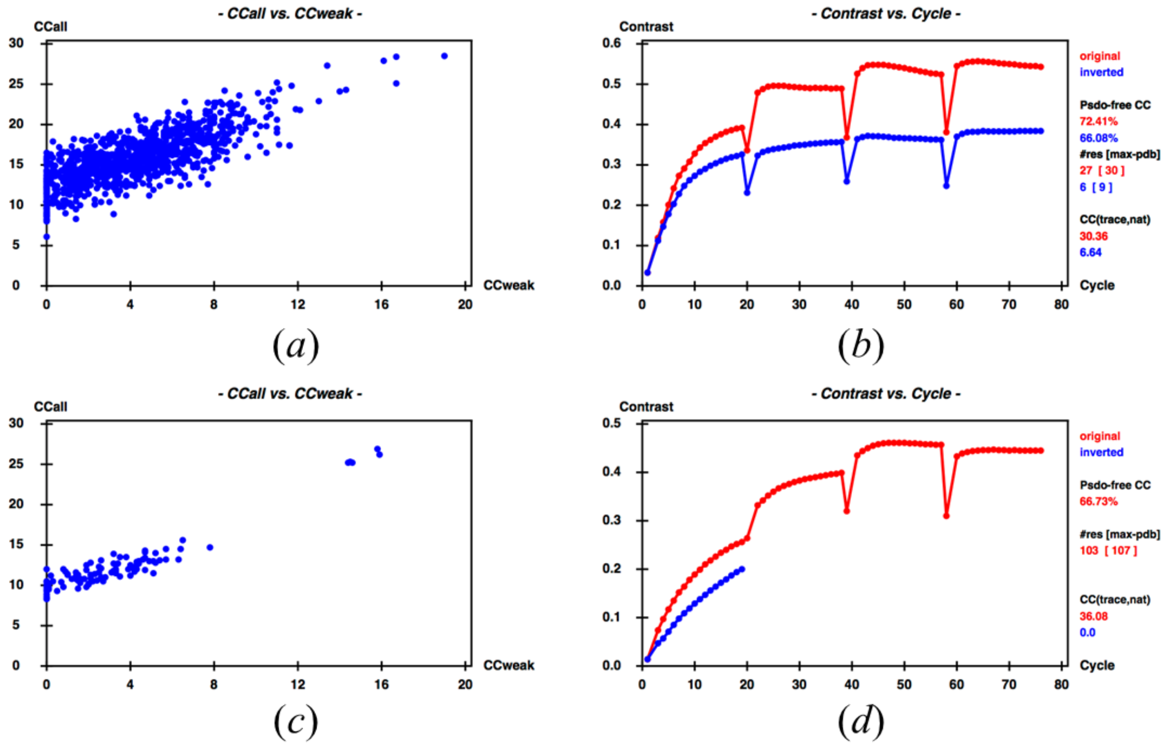

Figure S2 *SHELXD* and *SHELXE* plots. (*a*, *b*) Insulin sulfur SAD phasing. (*c*, *d*) Lysozyme bromine SAD phasing.

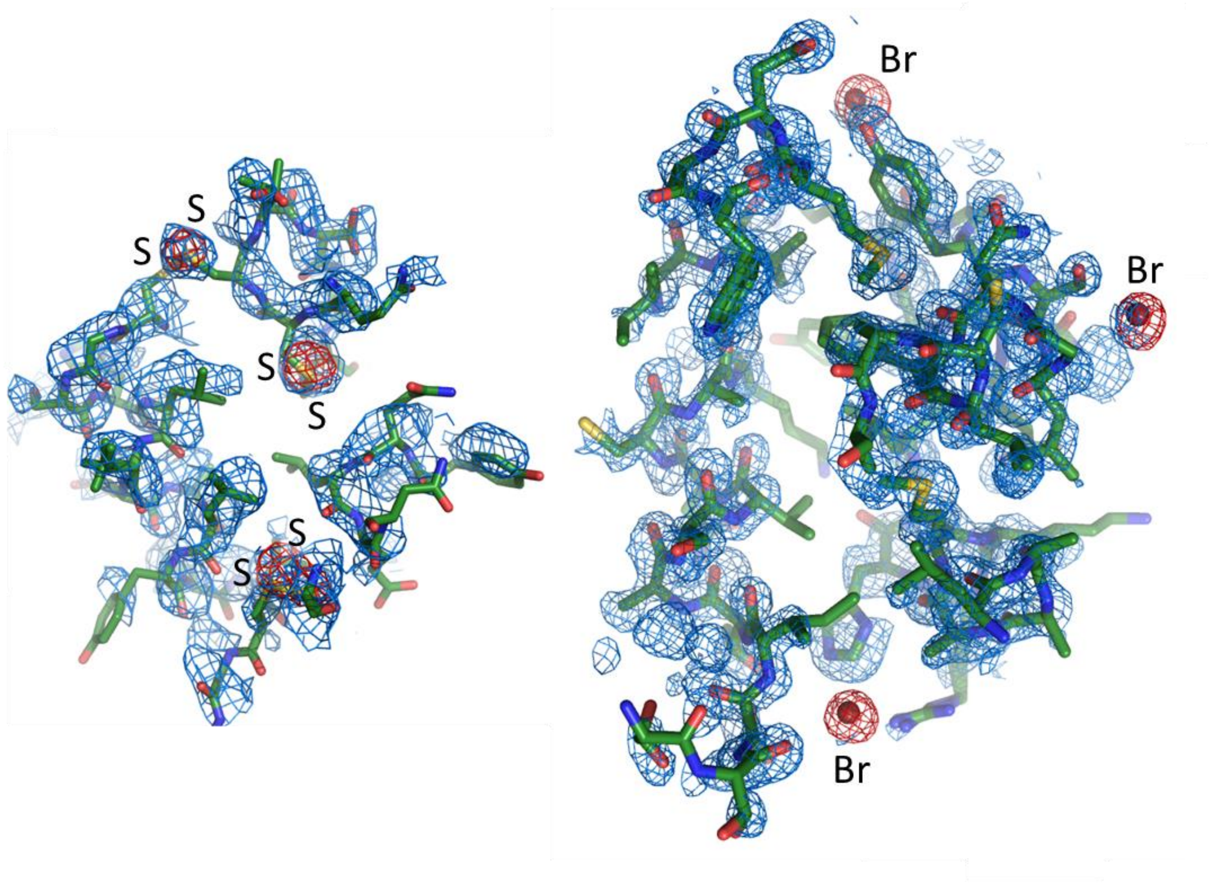

(*a*) (*b*)

Figure S3 The initial electron-density maps obtained by sulfur SAD (*a*) and bromine SAD (*b*) phasing for measurements made with insulin and lysozyme, respectively, by the IMISXcryo method at 100 K. Residues Gln5-Glu17 of chain A and residues Cys7-Gly20 of chain B in insulin (*a*) and residues Leu11-Ser33 and Ser86-Ala107 in lysozyme (*b*) are shown. The initial maps obtained after density modification with *SHELXE* were contoured at 1 σ and are shown as a blue mesh. The anomalous difference map contoured at 5 σ is shown as a red mesh. Sulfur and bromine atoms are labelled. The final model is shown in stick representation. The resolutions are 2.4 Å and 1.5 Å for the corresponding sulfur and bromine SAD data at 100 K.

Table S1 Comparison of structures obtained with IMISXcryo and loop-harvested samples at 100K

	**Insulin-N**		**Lyso-N**		**Lyso-Br**		**AlgE**		**$PepT_{St}$**		**β_2AR**		**DgkA**	
	**IMIXScryo**	**Loop**	**IMIXScryo**	**Loop 4xjd***	**IMIXScryo**	**Loop 4xjg***	**IMIXScryo**	**Loop 4xnl***	**IMIXScryo**	**Loop**	**IMIXScryo**	**Loop 2rh1***	**IMIXScryo**	**Loop**
**Res. (Å)**	1.5	1.5	1.7	1.8	1.5	1.8	2.4	2.9	2.4	2.4	2.5	2.4	2.8	2.8
**r.m.s.d.**	0.138<br>(51 residues, 203 atoms aligned)		0.351<br>(129 residues, 501 atoms aligned)		0.098<br>(129 residues, 483 atoms aligned)		0.599<br>(403 residues, 1,305 atoms aligned)		0.158<br>(448 residues, 1,568 atoms aligned)		0.200<br>(442 residues, 1,676 atoms aligned)		0.238<br>(585 residues, 2,012 atoms aligned)	
**Ligand**	None		None		None		None		1×Ala-Phe		1×carazolol		None	
**Lipid**	ND**		ND		ND		2×7.8MAG	5×7.8MAG	21×7.8MAG		3×cholesterol		11×7.8MAG	16×7.8MAG
**Non-protein molecules**	2×PO_4^{3-}<br>1×PEG400		1×Na^+<br>7×Br<br>1×Acetate<br>1×PEG400	7×Cl	1×Na^+<br>7×Br<br>1×Acetate<br>1×PEG400	5×Br	1×Ca^{2+}<br>2×Na^-<br>7×LDAO<br>2×MES<br>5×PEG400	8×LDAO<br>1×PEG400	1×PO_4^{3-}<br>2×PEG400		5×SO_4^{2-}<br>1×Acetamide<br>2×Butanediol<br>1×Maltose<br>1×Palmitoyl chain<br>1×PEG400		1×Zn^{2+}<br>1×Acetate<br>1×Citrate	ND

* Reference structures are from the literature and include PDB IDs 4xjd, 4xjg and 4xnl from Huang *et al*. (Huang *et al*., 2015) and2rh1 from Cherezov *et al*. (Cherezov *et al*., 2007).

** ND, none detected.

References

Cherezov, V., Rosenbaum, D. M., Hanson, M. A., Rasmussen, S. G., Thian, F. S., Kobilka, T. S., Choi, H. J., Kuhn, P., Weis, W. I., Kobilka, B. K. & Stevens, R. C. (2007). *Science*, **318**, 1258-1265.

Huang, C. Y., Olieric, V., Ma, P., Panepucci, E., Diederichs, K., Wang, M. & Caffrey, M. (2015). *Acta Cryst. D***71**, 1238-1256.

Supplementary Movies

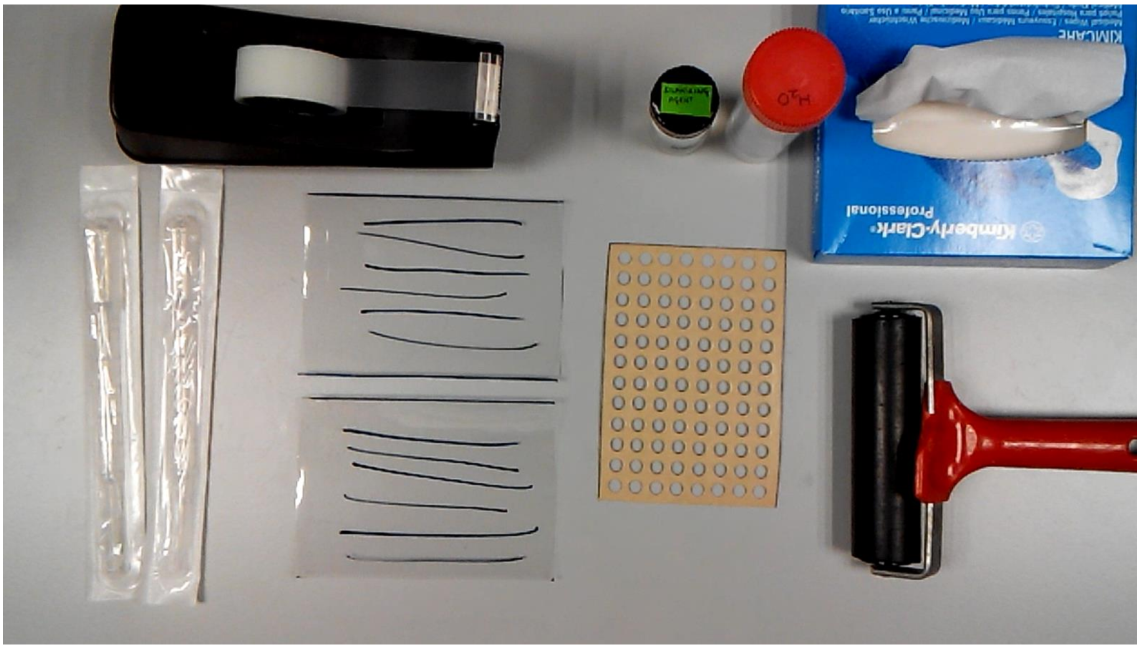

Still Image and Legend for Movie

Supplementary Movie S1

The movie shows the materials and the method used to set up and to use IMISX plates.

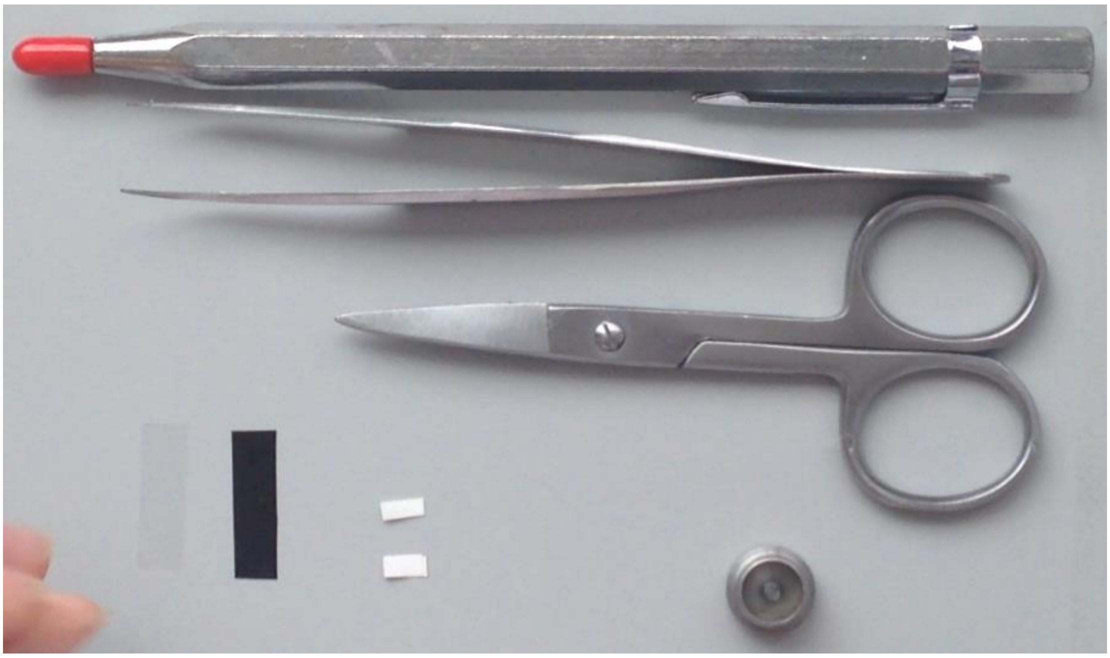

Still Image and Legend for Movie

Supplementary Movie S2

The movie shows the materials and the method used to assemble the composite support for the IMISX well.

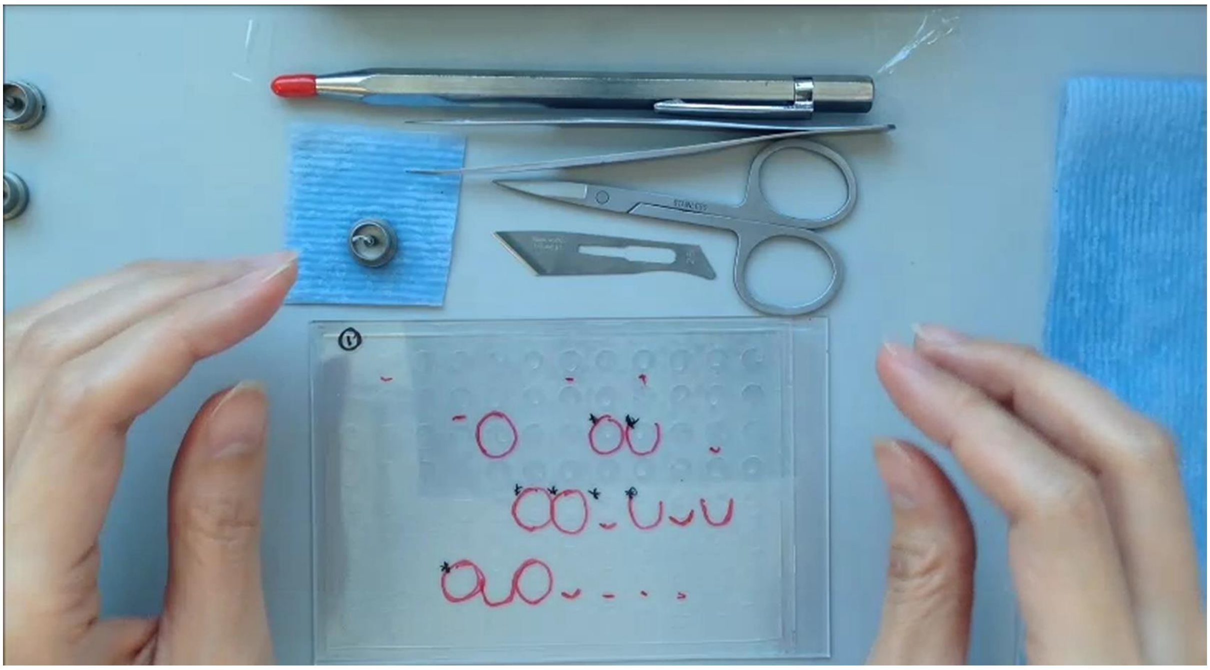

Still Image and Legend for Movie

Supplementary Movie S3

The movie shows the materials and the methods used to remove the well from an IMISX plate, to mount it on a goniometer base and to snap-cool the sample in liquid nitrogen.

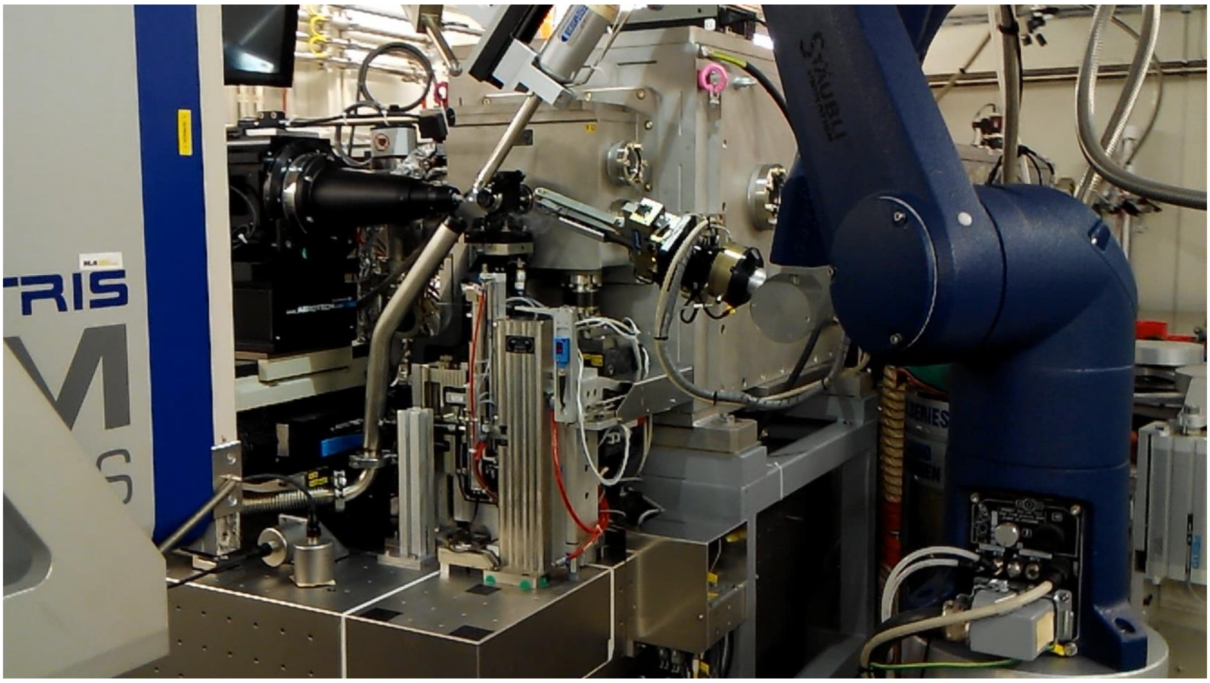

Still Image and Legend for Movie

Supplementary Movie S4

The movie shows the sample handling robot on beamline PX I at the SLS moving IMISXcryo samples between the Dewar and the goniometer.
